# Supplementary material for: Factors associated with tuberculosis treatment initiation among bacteriologically negative individuals evaluated for tuberculosis: An individual patient data meta-analysis
Source: PLoS Med. 2025 Jan 13;22(1):e1004502. doi: 10.1371/journal.pmed.1004502 (PMC11729971; doi:10.1371/journal.pmed.1004502)
Supplement: S1 Supplement — Table A. PRISMA Checklist. Table B. Demographic and clinical characteristics of participants, by study. Table C. Odds ratios of TB treatment initiation following negative diagnostic test result: country random effects from primary analysis. Table D. Odds ratios of TB treatment initiation following negative diagnostic test result: secondary analysis for data sets including chest X-ray results. Table E. Odds of TB treatment initiation following negative diagnostic test result: study random effects from alternative model specification. Table F. Sensitivity analysis comparing Logit vs. Probit model. Table G. Stratified analysis by diagnostic tests. Table H. Stratified analysis by HIV results. Table I. Odds ratios of TB treatment initiation following a negative diagnostic test result (Xpert/Xpert Ultra combined). Table J. Absolute risk and risk difference of treatment initiation. Table K. Contact information for accessing each data set included in the study. Text A. Search terms for Embase (Elsevier, embase.com). Text B. Hierarchical Bayesian logistic regression model. Text C. Description of individual studies included in analysis. (PDF) [file pmed.1004502.s001.pdf]

SUPPLEMENT

Table A. PRISMA Checklist

Section and Topic	Item #	Checklist item	Location where item is reported
**TITLE**			
Title	1	Identify the report as a systematic review.	Title
**ABSTRACT**			
Abstract	2	See the PRISMA 2020 for Abstracts checklist.	Abstract
**INTRODUCTION**			
Rationale	3	Describe the rationale for the review in the context of existing knowledge.	Introduction, Paragraph 2
Objectives	4	Provide an explicit statement of the objective(s) or question(s) the review addresses.	Introduction, Paragraph 3
**METHODS**			
Eligibility criteria	5	Specify the inclusion and exclusion criteria for the review and how studies were grouped for the syntheses.	Methods, Paragraph 2-3
Information sources	6	Specify all databases, registers, websites, organisations, reference lists and other sources searched or consulted to identify studies. Specify the date when each source was last searched or consulted.	Methods, Paragraph 2-3
Search strategy	7	Present the full search strategies for all databases, registers and websites, including any filters and limits used.	Supplement, page 12, search terms
Selection process	8	Specify the methods used to decide whether a study met the inclusion criteria of the review, including how many reviewers screened each record and each report retrieved, whether they worked independently, and if applicable, details of automation tools used in the process.	Methods, Paragraph 4
Data collection process	9	Specify the methods used to collect data from reports, including how many reviewers collected data from each report, whether they worked independently, any processes for obtaining or confirming data from study investigators, and if applicable, details of automation tools used in the process.	Methods, Paragraph 4
Data items	10a	List and define all outcomes for which data were sought. Specify whether all results that were compatible with each outcome domain in each study were sought (e.g. for all measures, time points, analyses), and if not, the methods used to decide which results to collect.	Methods, Paragraph 5-8
	10b	List and define all other variables for which data were sought (e.g. participant and intervention characteristics, funding sources). Describe any assumptions made about any missing or unclear information.	Methods, Paragraph 5-8
Study risk of bias assessment	11	Specify the methods used to assess risk of bias in the included studies, including details of the tool(s) used, how many reviewers assessed each study and whether they worked independently, and if applicable, details of automation tools used in the process.	Methods, Paragraph 11
Effect measures	12	Specify for each outcome the effect measure(s) (e.g. risk ratio, mean difference) used in the synthesis or presentation of results.	Methods, Paragraph 9-12
Synthesis methods	13a	Describe the processes used to decide which studies were eligible for each synthesis (e.g. tabulating the study intervention characteristics and comparing against the planned groups for each synthesis (item #5)).	Methods, Paragraph 9-12
	13b	Describe any methods required to prepare the data for presentation or synthesis, such as handling of missing summary statistics, or data conversions.	Methods, Paragraph 9-12
	13c	Describe any methods used to tabulate or visually display results of individual studies and syntheses.	Methods, Paragraph 9-12
	13d	Describe any methods used to synthesize results and provide a rationale for the choice(s). If meta-analysis was performed, describe the model(s), method(s) to identify the presence and extent of statistical heterogeneity, and software package(s) used.	Methods, Paragraph 9-12
	13e	Describe any methods used to explore possible causes of heterogeneity among study results (e.g. subgroup analysis, meta-regression).	Methods, Paragraph 10-12

Section and Topic	Item #	Checklist item	Location where item is reported
	13f	Describe any sensitivity analyses conducted to assess robustness of the synthesized results.	Methods, Paragraph 10-12
Reporting bias assessment	14	Describe any methods used to assess risk of bias due to missing results in a synthesis (arising from reporting biases).	Methods, Paragraph 12
Certainty assessment	15	Describe any methods used to assess certainty (or confidence) in the body of evidence for an outcome.	Methods, Paragraph 12
**RESULTS**			
Study selection	16a	Describe the results of the search and selection process, from the number of records identified in the search to the number of studies included in the review, ideally using a flow diagram.	Results, Fig 1
	16b	Cite studies that might appear to meet the inclusion criteria, but which were excluded, and explain why they were excluded.	Results, Fig 1, Table B (in S1)
Study characteristics	17	Cite each included study and present its characteristics.	Results, Table 1, Table B (in S1)
Risk of bias in studies	18	Present assessments of risk of bias for each included study.	Results, Table 3
Results of individual studies	19	For all outcomes, present, for each study: (a) summary statistics for each group (where appropriate) and (b) an effect estimate and its precision (e.g. confidence/credible interval), ideally using structured tables or plots.	Results, Table B
Results of syntheses	20a	For each synthesis, briefly summarise the characteristics and risk of bias among contributing studies.	Results, Table 1
	20b	Present results of all statistical syntheses conducted. If meta-analysis was done, present for each the summary estimate and its precision (e.g. confidence/credible interval) and measures of statistical heterogeneity. If comparing groups, describe the direction of the effect.	Results, Tables 2-3, Fig 2, Tables C-K
	20c	Present results of all investigations of possible causes of heterogeneity among study results.	Results, Tables G-H
	20d	Present results of all sensitivity analyses conducted to assess the robustness of the synthesized results.	Results, Tables E-F
Reporting biases	21	Present assessments of risk of bias due to missing results (arising from reporting biases) for each synthesis assessed.	Results, Tables E-H
Certainty of evidence	22	Present assessments of certainty (or confidence) in the body of evidence for each outcome assessed.	Included in all exhibits
**DISCUSSION**			
Discussion	23a	Provide a general interpretation of the results in the context of other evidence.	Discussion, Paragraph 1-7
	23b	Discuss any limitations of the evidence included in the review.	Discussion, Paragraph 8-11
	23c	Discuss any limitations of the review processes used.	Discussion, Paragraph 8-11
	23d	Discuss implications of the results for practice, policy, and future research.	Discussion, Paragraph 12
**OTHER INFORMATION**			
Registration and protocol	24a	Provide registration information for the review, including register name and registration number, or state that the review was not registered.	Methods, Paragraph 1
	24b	Indicate where the review protocol can be accessed, or state that a protocol was not prepared.	Methods, Paragraph 1
	24c	Describe and explain any amendments to information provided at registration or in the protocol.	Methods, Paragraph 1
Support	25	Describe sources of financial or non-financial support for the review, and the role of the funders or sponsors in the review.	Funding section
Competing interests	26	Declare any competing interests of review authors.	Competing interests section

Section and Topic	Item #	Checklist item	Location where item is reported
Availability of data, code and other materials	27	Report which of the following are publicly available and where they can be found: template data collection forms; data extracted from included studies; data used for all analyses; analytic code; any other materials used in the review.	Data availability section, Table K

Table B. Demographic and clinical characteristics of participants, by study.

	Churchyard et al., 2015	Penn-Nicholson et al., 2021	Dorman et al., unpublished	Dorman et al., 2018	Theron et al., unpublished	Mupfumi et al., 2014
Study sites eligible for inclusion*	South Africa	Ethiopia, India, Papua New Guinea, and Peru	Kenya, South Africa, and Uganda	Belarus, Georgia, India, and South Africa	South Africa	Zimbabwe
Analytic population, n	4001	1411	906	870	777	390
Individuals with treatment initiation, n (primary outcome)	130	22	61	23	4	66
Alternative outcome, n	20	19	61	23	1	16
Age category, n						
18 – 30 years	1322	370	263	206	340	80
31 – 40 years	1134	275	282	214	267	174
41 years and above	1545	766	361	450	170	136
Male, n	1444	784	459	522	300	171
Individuals with history of prior TB, n	613	192	223	255	107	49
Reported cough						
None	759	0	0	48	566	200
Yes	3242	1411	906	822	211	190
Reported night sweats						
None	2254	829	310	433	618	188
Yes	1747	582	596	437	159	202
Reported fever						
None	2219	629	275	360	742	161
Yes	1782	782	631	510	35	229
HIV						
Negative	1203	703	429	318	0	0
Positive, not on ART	1246	0	64	100	0	61
Positive, on ART	624	28	412	67	777	329
Unknown	928	680	1	385	0	0
Individuals with abnormal chest X-ray result, n	NA	196	NA	250	NA	NA
Year of data collection	2012	2019 – 2020	2018 – 2020	2016	2017 – 2020	2011 – 2012
Initial TB tests used for diagnoses**	SSM, Xpert	Xpert, Xpert Ultra	Xpert, Xpert Ultra	Xpert	Xpert Ultra	SSM, Xpert

	Pereira et al., 2020	Hanrahan et al., 2015	Mishra et al., 2020	Luetkemeyer et al. 2016	Bjerrum et al., 2020	Agizew et al., 2019
Study sites eligible for inclusion*	Brazil	South Africa	South Africa	Brazil, South Africa	Ghana	Botswana
Analytic population, n	147	168	212	280	121	5838
Individuals with treatment initiation, n (primary outcome)	0	5	0	28	6	132
Alternative outcome	0	2	0	28	2	33
Age category, n						
18 – 30 years	24	25	68	54	33	2099
31 – 40 years	12	66	48	92	42	2248
41 years and above	111	77	96	134	46	1491
Male, n	76	120	113	133	38	1921
Individuals with history of prior TB, n	0	0	87	36	9	624
Reported cough						
None	0	22	12	15	66	4793
Yes	147	146	200	265	55	1045
Reported night sweats						
None	0	128	62	116	82	5321
Yes	147	40	150	164	39	517
Reported fever						
None	49	132	186	134	60	5340
Yes	98	36	26	146	61	498
HIV						
Negative	144	24	170	110	0	0
Positive, not on ART	3	38	42	170	38	5838
Positive, on ART	0	104	0	0	83	0
Unknown	0	2	0	0	0	0
Individuals with abnormal chest X-ray result, n	NA	10	NA	NA	NA	NA
Year of data collection	2018 – 2019	2011 – 2013	2016 – 2018	2012 – 2013	2013 – 2014	2012 – 2014
Initial TB tests used for diagnoses**	Xpert Ultra	Xpert	SSM, Xpert, Xpert Ultra	SSM	Xpert	SSM. Xpert

Table B (continued). Demographic and clinical characteristics of participants, by study.

* Data from countries with high income and low TB burdens were not considered for inclusion.
** Diagnostic tests performed solely for research purposes, which were not part of routine practice in the setting, were excluded.

Table C. Odds ratios of TB treatment initiation following negative diagnostic test result: country random effects from primary analysis.

Country	Adjusted odds ratio of treatment initiation, compared to cross-country average (95% Credible Intervals)
Belarus	0.56 (0.12, 2.04)
Botswana	0.55 (0.26, 1.17)
Brazil	0.33 (0.12, 0.83)
Ethiopia	0.57 (0.16, 1.80)
Georgia	0.48 (0.17, 1.17)
Ghana	1.15 (0.42, 3.00)
India	0.97 (0.46, 2.12)
Kenya	2.14 (0.92, 5.09)
Papua New Guinea	1.34 (0.42, 3.93)
Peru	0.95 (0.31, 2.68)
South Africa	0.36 (0.18, 0.72)
Uganda	7.01 (3.43, 15.16)
Zimbabwe	2.88 (1.32, 6.34)

Table D. Odds ratios of TB treatment initiation following negative diagnostic test result: secondary analysis for datasets including chest x-ray results.

	Adjusted odds ratio of treatment initiation (95% Credible Intervals)
Age category	
18 – 30 years	Ref
31 – 40 years	1.30 (0.53, 3.26)
41 years and above	1.47 (0.71, 3.25)
Sex	
Female	Ref
Male	1.00 (0.56, 1.83)
History of prior TB	
None	Ref
Yes	0.64 (0.28, 1.39)
Unknown	0.50 (0.10, 1.91)
Reported cough	
None	Ref
Yes	1.26 (0.22, 9.67)
Reported night sweats	
None	Ref
Yes	1.14 (0.60, 2.18)
Reported fever	
None	Ref
Yes	0.68 (0.36, 1.27)
HIV	
Negative	Ref
Positive, not on ART	1.03 (0.17, 5.44)
Positive, on ART	1.28 (0.27, 5.51)
Unknown	0.58 (0.27, 1.15)
Chest X-ray	
Normal	Ref
Abnormal	6.89 (3.29, 14.42)
Unknown	0.69 (0.16, 2.54)
Diagnostic test	
Sputum Smear	-
Xpert	Ref
Xpert Ultra	0.77 (0.05, 16.03)
Year	0.86 (0.66, 1.11)

Table E. Odds of TB treatment initiation following negative diagnostic test result: study random effects from alternative model specification.

Study	Adjusted odds ratio of treatment initiation, compared to cross-study average (95% Credible Intervals)
Churchyard et al., 2015	0.53 (0.20, 1.50)
Penn-Nicholson et al., 2021	0.85 (0.27, 2.57)
Dorman et al., unpublished	3.77 (1.30, 11.00)
Dorman et al., 2018	0.70 (0.28, 1.77)
Theron et al., unpublished	1.14 (0.30, 4.05)
Mupfumi et al., 2014	4.97 (1.76, 15.56)
Pereira et al., 2020	0.27 (0.02, 1.67)
Hanrahan et al., 2015	1.30 (0.25, 9.72)
Mishra et al., 2020	0.10 (0.01, 0.48)
Luetkemeyer et al. 2016	1.18 (0.45, 3.21)
Bjerrum et al., 2020	1.68 (0.51, 5.79)
Agizew et al., 2019	0.87 (0.34, 2.33)

Table F. Sensitivity analysis comparing Logit vs. Probit model*.

	Main analysis (logit)			Probit model		
	Estimate	Standard Error	t-ratio	Estimate	Standard Error	t-ratio
Age category						
18 – 30 years	Ref			Ref		
31 – 40 years	0.16	0.13	1.23	0.07	0.06	1.31
41 years and above	0.10	0.13	0.78	0.04	0.06	0.67
Sex						
Female	Ref			Ref		
Male	0.48	0.10	4.85	0.21	0.04	4.61
History of prior TB						
None	Ref			Ref		
Yes	0.31	0.12	2.61	0.14	0.06	2.53
Unknown	-0.30	0.44	-0.68	-0.10	0.18	-0.58
Reported cough						
None	Ref			Ref		
Yes	1.53	0.15	10.04	0.68	0.07	10.23
Reported night sweats						
None	Ref			Ref		
Yes	0.41	0.11	3.69	0.19	0.05	3.74
Reported fever						
None	Ref			Ref		
Yes	0.12	0.11	1.10	0.05	0.05	1.02
HIV						
Negative	Ref			Ref		
Positive, not on ART	0.52	0.17	3.14	0.24	0.08	3.20
Positive, on ART	-0.10	0.18	-0.57	-0.02	0.09	-0.29
Unknown	-0.20	0.20	-1.02	-0.07	0.08	-0.83
Diagnostic test						
Sputum Smear	Ref			Ref		
Xpert	-0.27	0.12	-2.31	-0.12	0.05	-2.29
Xpert Ultra	-0.56	0.31	-1.78	-0.19	0.15	-1.25
Year	-0.20	0.05	-4.14	-0.09	0.02	-4.07

* The expected log pointwise predictive density (ELPD) difference of 3.6 with a standard error of 64.5 suggests that the main analysis (logit model) has a slightly better fit. However, the difference between the two models is not statistically significant.

Table G. Stratified analysis by diagnostic tests.

	Main analysis (95% Credible Intervals)	SSM (95% Credible Intervals)	Xpert + Xpert Ultra (95% Credible Intervals)
Age category			
18 – 30 years	Ref	Ref	Ref
31 – 40 years	1.17 (0.92, 1.51)	1.10 (0.73, 1.64)	1.24 (0.90, 1.73)
41 years and above	1.11 (0.87, 1.43)	0.80 (0.54, 1.22)	1.35 (0.98, 1.86)
Sex			
Female	Ref	Ref	Ref
Male	1.61 (1.31, 1.95)	1.80 (1.33, 2.46)	1.48 (1.15, 1.91)
History of prior TB			
None	Ref	Ref	Ref
Yes	1.36 (1.06, 1.73)	1.50 (1.01, 2.17)	1.29 (0.95, 1.76)
Unknown	0.73 (0.28, 1.65)	.	0.81 (0.30, 1.86)
Reported cough			
None	Ref	Ref	Ref
Yes	4.62 (3.42, 6.27)	4.48 (2.78, 7.46)	4.50 (1.10, 1.93)
Reported night sweats			
None	Ref	Ref	Ref
Yes	1.50 (1.21, 1.90)	1.60 (1.13, 2.28)	1.45 (1.10, 1.93)
Reported fever			
None	Ref	Ref	Ref
Yes	1.13 (0.91, 1.39)	1.00 (0.72, 1.40)	1.22 (0.93, 1.60)
HIV			
Negative	Ref	Ref	Ref
Positive, not on ART	1.68 (1.23, 2.32)	1.74 (1.10, 2.84)	1.63 (1.10, 2.61)
Positive, on ART	0.90 (0.64, 1.30)	1.04 (0.54, 1.92)	0.88 (0.55, 1.36)
Unknown	0.82 (0.55, 1.20)	1.16 (0.61, 2.13)	0.65 (0.39, 1.08)
Diagnostic test			
Sputum Smear	Ref	.	.
Xpert	0.77 (0.62, 0.96)	.	Ref
Xpert Ultra	0.57 (0.30, 1.07)	.	0.79 (0.44, 1.44)
Year	0.81 (0.74, 0.90)	0.77 (0.62, 0.93)	0.81 (0.71, 0.91)

Table H. Stratified analysis by HIV results*.

	Main analysis (95% Credible Intervals)	HIV negative	HIV Positive, not on ART	HIV Positive, on ART
Age category				
18 – 30 years	Ref	Ref	Ref	Ref
31 – 40 years	1.17 (0.92, 1.51)	1.81 (0.97, 3.36)	1.10 (0.77, 1.54)	0.86 (0.49, 1.58)
41 years and above	1.11 (0.87, 1.43)	1.65 (0.95, 2.90)	1.11 (0.78, 1.58)	0.85 (0.48, 1.55)
Sex				
Female	Ref	Ref	Ref	Ref
Male	1.61 (1.31, 1.95)	2.03 (1.29, 3.28)	1.66 (1.25, 2.21)	1.60 (1.03, 2.45)
History of prior TB				
None	Ref	Ref	Ref	Ref
Yes	1.36 (1.06, 1.73)	1.72 (1.04, 2.85)	1.17 (0.81, 1.71)	1.62 (0.98, 2.71)
Unknown	0.73 (0.28, 1.65)	0.34 (0.02, 2.40)	1.70 (0.38, 5.71)	0.91 (0.13, 4.10)
Reported cough				
None	Ref	Ref	Ref	Ref
Yes	4.62 (3.42, 6.27)	14.24 (2.07, 305.0)	5.49 (3.87, 7.83)	2.13 (1.22, 3.79)
Reported night sweats				
None	Ref	Ref	Ref	Ref
Yes	1.50 (1.21, 1.90)	1.66 (1.06, 2.65)	1.36 (0.97, 1.93)	1.67 (1.06, 2.67)
Reported fever				
None	Ref	Ref	Ref	Ref
Yes	1.13 (0.91, 1.39)	0.64 (0.41, 1.00)	1.63 (1.19, 2.26)	1.13 (0.73, 1.77)
HIV				
Negative	Ref	.	.	.
Positive, not on ART	1.68 (1.23, 2.32)	.	.	.
Positive, on ART	0.90 (0.64, 1.30)	.	.	.
Unknown	0.82 (0.55, 1.20)	.	.	.
Diagnostic test				
Sputum Smear	Ref	Ref	Ref	Ref
Xpert	0.77 (0.62, 0.96)	0.53 (0.25, 1.03)	0.80 (0.58, 1.09)	0.58 (0.33, 1.01)
Xpert Ultra	0.57 (0.30, 1.07)	0.62 (0.18, 2.09)	0.85 (0.19, 3.49)	0.30 (0.10, 0.89)
Year	0.81 (0.74, 0.90)	0.57 (0.44, 0.72)	0.90 (0.76, 1.05)	0.98 (0.81, 1.16)

*HIV Unknown stratum effective sample sizes were too low, indicating posterior means and medians may be unreliable.

Table I. Odds ratios of TB treatment initiation following a negative diagnostic test result (Xpert/ Xpert Ultra combined).

	Univariable analysis (95% Credible Intervals)	Multivariable analysis* (95% Credible Intervals)
Age category		
18 – 30 years	Ref	Ref
31 – 40 years	1.44 (1.13, 1.83)	1.17 (0.91, 1.53)
41 years and above	1.42 (1.12, 1.80)	1.10 (0.86, 1.43)
Sex		
Female	Ref	Ref
Male	1.80 (1.50, 2.16)	1.61 (1.33, 1.96)
History of prior TB		
None	Ref	Ref
Yes	1.61 (1.13, 2.03)	1.37 (1.08, 1.74)
Unknown	0.81 (0.32, 1.77)	0.73 (0.28, 1.69)
Reported cough		
None	Ref	Ref
Yes	5.93 (4.52, 7.88)	4.60 (3.44, 6.18)
Reported night sweats		
None	Ref	Ref
Yes	2.34 (1.89, 2.89)	1.51 (1.21, 1.89)
Reported fever		
None	Ref	Ref
Yes	1.84 (1.48, 2.28)	1.13 (0.91, 1.39)
HIV		
Negative	Ref	Ref
Positive, not on ART	1.88 (1.38, 2.57)	1.70 (1.23, 2.33)
Positive, on ART	0.73 (0.52, 1.02)	0.90 (0.63, 1.28)
Unknown	0.98 (0.65, 1.46)	0.82 (0.54, 1.22)
Diagnostic test		
Sputum Smear	Ref	Ref
Xpert or Xpert Ultra	0.56 (0.45, 0.69)	0.77 (0.61, 0.96)
Year	0.77 (0.71, 0.83)	0.80 (0.73, 0.87)

*Multivariable regression model also included country random effects, Ref = reference category.

Table J. Absolute risk and risk difference of treatment initiation. *

	Absolute risk (%) of treatment initiation (95% Credible Intervals)	Risk difference** (95% Credible Intervals)	Relative change (%) in probability of treatment initiation
Age category			
18 – 30 years	2.89 (0.11, 12.67)	Ref	Ref
31 – 40 years	3.35 (0.13, 14.47)	0.45 (-0.19, 2.41)	17.7 (-7.7, 50.2)
41 years and above	3.17 (0.12, 13.77)	0.28 (-0.41, 1.81)	11.2 (-12.6, 40.9)
Sex			
Female	2.55 (0.10, 10.56)	Ref	Ref
Male	3.93 (0.16, 15.96)	1.39 (0.06, 5.55)	59.3 (31.5, 91.2)
History of prior TB			
None	3.00 (0.12, 12.87)	Ref	Ref
Yes	3.99 (0.16, 16.93)	0.99 (0.03, 4.39)	35.9 (7.0, 70.8)
Unknown	2.43 (0.09, 11.48)	-0.57 (-4.27, 1.74)	-19.2(-70.2, 57.5)
Reported cough			
None	1.17 (0.08, 4.52)	Ref	Ref
Yes	4.90 (0.39, 17.80)	3.73 (0.30, 13.32)	348.7 (231.1, 497.2)
Reported night sweats			
None	2.65 (0.12, 10.96)	Ref	Ref
Yes	3.84 (0.17, 15.60)	1.19 (0.06, 4.84)	49.0 (20.7, 82.6)
Reported fever			
None	3.00 (0.12, 12.96)	Ref	Ref
Yes	3.35 (0.14, 14.35)	0.35 (-0.22, 1.93)	13.0 (-8.1, 37.0)
HIV			
Negative	2.69 (0.13, 12.08)	Ref	Ref
Positive, not on ART	4.29 (0.23, 18.64)	1.60 (0.08, 6.81)	67.6 (20.7, 126.9)
Positive, on ART	2.46 (0.12, 11.05)	-0.24 (-1.91, 0.74)	-8.0 (-36.5, 28.9)
Unknown	2.24 (0.11, 10.16)	-0.45 (-2.61, 0.41)	-16.7 (-44.3, 20.4)
Diagnostic test			
Sputum Smear	3.75 (0.21, 16.96)	Ref	Ref
Xpert	2.95 (0.16, 13.54)	-0.80 (-3.66, -0.02)	-22.0 (-37.0, -3.2)
Xpert Ultra	2.32 (0.12, 11.16)	-1.43 (-6.80, 0.14)	-38.9 (-68.3, 8.8)

* The values are derived using posterior draws of predicted probabilities from the main regression model (Table 2).
** The units are percentage points, Ref = reference category.

Table K. Contact information for accessing each dataset included in the study.

Study	Contact information for data access*
Churchyard et al., 2015	katherine.fielding@lshtm.ac.uk
Penn-Nicholson et al., 2021	mikashmi.kohli@finddx.org
Dorman et al., unpublished	dorman@musc.edu
Dorman et al., 2018	dorman@musc.edu
Theron et al., unpublished	gtheron@sun.ac.za
Mupfumi et al., 2014	lmupfumi@gmail.com
Pereira et al., 2020	drsilva@hcpa.edu.br
Hanrahan et al., 2015	chanrah1@jhmi.edu
Mishra et al., 2020	gtheron@sun.ac.za
Luetkemeyer et al. 2016	annie.luetkemeyer@ucsf.edu
Bjerrum et al., 2020	steph@medicinsk.dk
Agizew et al., 2019	tefera.agizew@apopo.org

*Subject to IRB approval and data use agreement.
The Institutional Review Board of the Harvard T.H. Chan School of Public Health: irb@hsph.harvard.edu

Text A. Search terms for Embase (Elsevier, embase.com).

Advanced Search:

Source: Embase subset (from 1974 to present)

Date: Publication years from 2010 - 2022

1) 'tuberculosis'/de OR 'lung tuberculosis'/de OR tuberculosis:ab,ti,kw

2) 'molecular diagnostics'/de OR 'molecular diagnosis'/de OR 'Mycobacterium tuberculosis test kit'/de OR 'polymerase chain reaction system'/de OR 'xpert'/de OR 'xpert ultra'/de OR 'xpert mtb rif ultra'/de OR 'sputum analysis'/de OR ('sputum smear'/de AND 'microscopy'/exp) OR 'sputum culture'/de OR genexpert:ab,ti,kw OR xpert:ab,ti,kw OR 'smear microscopy':ab,ti,kw OR 'sputum microscopy':ab,ti,kw

3) 'clinical trial'/de OR 'controlled clinical trial'/de OR 'randomized controlled trial'/exp OR 'diagnostic accuracy'/de OR 'diagnostic test accuracy study'/de OR 'evaluation study'/de OR 'clinical trial':ab,ti,kw OR random*:ab,ti,kw OR accuracy:ti,ab,kw OR evaluation:ab,ti,kw

1 AND 2 AND 3

NOT ('chapter'/it OR 'conference abstract'/it OR 'conference paper'/it OR 'conference review'/it OR 'editorial'/it OR 'review'/it)

Search terms for MEDLINE/PubMed (National Library of Medicine, NCBI)

("Tuberculosis"[Mesh:NoExp] OR "Tuberculosis, Pulmonary"[Mesh] OR tuberculosis[tiab]) AND ("Molecular Diagnostic Techniques"[Mesh:NoExp] OR ("Sputum"[Mesh] AND "Microscopy"[Mesh]) OR genexpert[tiab] OR xpert[tiab] OR smear microscopy[tiab] OR sputum microscopy[tiab]) AND ("randomized controlled trial"[pt] OR "controlled clinical trial"[pt] OR "random allocation"[mesh] OR "clinical trial"[pt] OR "evaluation study"[pt] OR "clinical trial"[tiab] OR random*[tiab] OR accuracy [tiab] OR evaluation[tiab]) AND 2010[pdat]: 2022[pdat].

Text B. Hierarchical Bayesian logistic regression model.

For the main analysis we fit the following regression model:

$$TREAT \sim Binomial\ (n = trials(1), p)$$

$$logit\ (p) = \beta_0 + \beta_{Age\_cat}Age_{cat} + \beta_{Sex}Sex + \beta_{TB_{hist}}TB_{hist} + \beta_{cough}Cough + \beta_{sweat}Sweat + \beta_{Fever}Fever + \beta_{HIV}HIV + \beta_{Diag_{test}}Diag_{test} + \beta_{year}Year + b_{country}$$

Where:

- $TREAT$ is the binary outcome variable indicating whether or not an individual initiated TB treatment.
- p is the probability of receiving treatment.
- Age_{cat} , $Sex, TB_{hist}, Cough, Sweat, Fever, HIV, Diag_{test}$, and $Year$ are exposure variables as defined in Table 2.
- $b_{country}$ represents the country random effect term.

All analyses were performed using the “brms” package (v.2.19.0) in R (v.4.2.3). We adopted the default prior distributions available in this package, with all main effects given prior student-*t* distributions with 3 degrees of freedom and a scale parameter of 10, and the random effects standard deviation given a half student-*t* prior with 3 degrees of freedom. The approach uses an extension of Hamiltonian Monte Carlo to sample from the posterior distribution of the regression model parameters (1–3). During the sampling process we ran 4 chains for 2000 samples each, retaining the last 1000 samples of each chain to produce a final set of 4,000 posterior samples. Convergence was assessed using Gelman-Rubin statistic (i.e., potential scale reduction factor (PSRF)), and by reviewing trace plots. PSRF values for all parameters were observed to be

1.00, confirming convergence (4), and trace-plots indicated chains were fully mixed. We also evaluated the effective sample sizes (ESS) for all our models. Bulk ESS and Tail ESS quantify sampling efficiency in the bulk of the posterior distribution and the tails of this distribution, respectively. The ESS values for each coefficient estimate were consistently above 1000, indicating that the sampling process was efficient and that our posterior samples are reliable. The reported coefficient estimates (aOR) in **Table 2** represent the posterior means for each parameter, derived by exponentiating regression outputs, along with equal-tailed 95% credible intervals (95% CI). Secondary analyses and alternative model specifications were performed using a similar approach.

Text C. Description of individual studies included in analysis.

The XTEND study was a pragmatic cluster-randomized trial conducted in South Africa assessing 6-month mortality of clinic attendees randomly allocated testing with Xpert MTB/RIF or sputum smear microscopy (5). Penn-Nicholson et al. conducted a prospective multi-center diagnostic accuracy study conducted in four countries (Peru, India, Ethiopia and Papua New Guinea) to assess the performance of the Truenat TB assays compared to Xpert MTB/RIF (6). Dorman et al. conducted a prospective multi-center diagnostic accuracy study in eight countries (South Africa, India, Georgia, and Belarus) assessing sensitivity and specificity of Xpert Ultra compared to Xpert (7). Dorman et al. subsequently conducted the Ultra 2 study in three countries (South Africa, Uganda, Kenya), incorporating the same study procedures as used in the Ultra study (7), with improvement in stability of the initial Ultra assay (Dorman et al., unpublished). Theron and the study team conducted a prospective diagnostic accuracy study in South Africa focusing on people living with HIV (PLHIV) attending clinics to start antiretroviral therapy (ART). The preliminary findings were published in the Union Conference. Mupfumi et al. conducted a pragmatic randomized control trial in Zimbabwe, examining the impact of Xpert on ART-associated TB and patient outcomes (8). Pereira et al assessed the diagnostic accuracy of Xpert Ultra in Brazil (9). Hanrahan et al. conducted a prospective cohort study in South Africa to investigate the effects of placement of Xpert at the point of care (POC) (10). Mishra et al. conducted a two-cohort diagnostic accuracy study in South Africa (11). Luetkemeyer et al. conducted a longitudinal multicenter study in the US, Brazil and South Africa to evaluate the Xpert assay (12). Bjerrum et al. conducted a diagnostic accuracy study of LAM studies among PLHIV in Ghana (13). Lastly, Agizew et al. conducted a stepped-wedge cluster randomized trial comparing TB treatment outcomes of SSM and Xpert among PLHIV in Botswana (14).

REFERENCES

1. Bürkner PC. brms: An R Package for Bayesian Multilevel Models Using Stan. Journal of Statistical Software. 2017 Aug 29;80:1–28.

2. Bürkner PC. Advanced Bayesian Multilevel Modeling with the R Package brms. The R Journal. 2018;10(1):395–411.

3. Bürkner PC. Bayesian Item Response Modeling in R with brms and Stan. Journal of Statistical Software. 2021 Nov 30;100:1–54.

4. Gelman A, Rubin DB. Inference from Iterative Simulation Using Multiple Sequences. Statistical Science. 1992 Nov;7(4):457–72.

5. Churchyard GJ, Stevens WS, Mametja LD, McCarthy KM, Chihota V, Nicol MP, et al. Xpert MTB/RIF versus sputum microscopy as the initial diagnostic test for tuberculosis: a cluster-randomised trial embedded in South African roll-out of Xpert MTB/RIF. Lancet Glob Health. 2015 Aug;3(8):e450–7.

6. Penn-Nicholson A, Gomathi SN, Ugarte-Gil C, Meaza A, Lavu E, Patel P, et al. A prospective multicentre diagnostic accuracy study for the Truenat tuberculosis assays. European Respiratory Journal [Internet]. 2021 Nov 1 [cited 2022 Mar 17];58(5). Available from: https://erj.ersjournals.com/content/58/5/2100526

7. Dorman SE, Schumacher SG, Alland D, Nabeta P, Armstrong DT, King B, et al. Xpert MTB/RIF Ultra for detection of Mycobacterium tuberculosis and rifampicin resistance: a prospective multicentre diagnostic accuracy study. Lancet Infect Dis. 2018 Jan;18(1):76–84.

8. Mupfumi L, Makamure B, Chirehwa M, Sagonda T, Zinyowera S, Mason P, et al. Impact of Xpert MTB/RIF on Antiretroviral Therapy-Associated Tuberculosis and Mortality: A Pragmatic Randomized Controlled Trial. Open Forum Infect Dis. 2014 Mar;1(1):ofu038.

9. Pereira GR, Barbosa MS, Dias NJD, dos Santos F de F, Rauber KA, Silva DR. Evaluation of Xpert MTB/RIF Ultra performance for pulmonary tuberculosis (TB) diagnosis in a city with high TB incidence in Brazil. Respiratory Medicine. 2020 Feb 1;162:105876.

10. Hanrahan CF, Clouse K, Bassett J, Mutunga L, Selibas K, Stevens W, et al. The patient impact of point-of-care vs. laboratory placement of Xpert® MTB/RIF. Int J Tuberc Lung Dis. 2015 Jul;19(7):811–6.

11. Mishra H, Reeve BWP, Palmer Z, Caldwell J, Dolby T, Naidoo CC, et al. Xpert MTB/RIF Ultra and Xpert MTB/RIF for diagnosis of tuberculosis in an HIV-endemic setting with a high burden of previous tuberculosis: a two-cohort diagnostic accuracy study. Lancet Respir Med. 2020 Apr;8(4):368–82.

12. Luetkemeyer AF, Firnhaber C, Kendall MA, Wu X, Mazurek GH, Benator DA, et al. Evaluation of Xpert MTB/RIF Versus AFB Smear and Culture to Identify Pulmonary Tuberculosis in Patients With Suspected Tuberculosis From Low and Higher Prevalence Settings. Clinical Infectious Diseases: An Official Publication of the Infectious Diseases Society of America. 2016 May 5;62(9):1081.

13. Bjerrum S, Broger T, Székely R, Mitarai S, Opintan JA, Kenu E, et al. Diagnostic Accuracy of a Novel and Rapid Lipoarabinomannan Test for Diagnosing Tuberculosis Among People With Human Immunodeficiency Virus. Open Forum Infect Dis. 2019 Dec 21;7(1):ofz530.

14. Agizew T, Boyd R, Auld AF, Payton L, Pals SL, Lekone P, et al. Treatment outcomes, diagnostic and therapeutic impact: Xpert vs. smear. A systematic review and meta-analysis. Int J Tuberc Lung Dis. 2019 Jan 1;23(1):82–92.
