# Supplementary material for: Factors associated with tuberculosis treatment initiation among bacteriologically negative individuals evaluated for tuberculosis: An individual patient data meta-analysis
Source: PLoS Med. 2025 Jan 13;22(1):e1004502. doi: 10.1371/journal.pmed.1004502 (PMC11729971; doi:10.1371/journal.pmed.1004502)
Supplement: S1 PROSPERO Protocol — (PDF) [file pmed.1004502.s002.pdf]

Factors associated with TB treatment initiation among bacteriologically-negative TB suspects: an individual patient data meta-analysis

To enable PROSPERO to focus on COVID-19 submissions, this registration record has undergone basic automated checks for eligibility and is published exactly as submitted. PROSPERO has never provided peer review, and usual checking by the PROSPERO team does not endorse content. Therefore, automatically published records should be treated as any other PROSPERO registration. Further detail is provided here.

Citation

Sun Kim, Melike Can, Tefera Agizew, Andrew Auld, Maria Balcells, Stephanie Bjerrum, Keertan Dheda, Susan Dorman, Aliasgar Esmail, Katherine Fielding, Alberto Garcia-Basteiro, Colleen Hanrahan, Wakjira Kebede, Mikashmi Kohli, Anne Luetkemeyer, Carol Mita, Byron Reeve, Denise Silva, Sedona Sweeney, Grant Theron, Anete Trajman, Anna Vassall, Joshua Warren, Marcel Yotebieng, Ted Cohen, Nick Menzies. Factors associated with TB treatment initiation among bacteriologically-negative TB suspects: an individual patient data meta-analysis. PROSPERO 2022 CRD42022287613 Available from: https://www.crd.york.ac.uk/prospero/display_record.php?ID=CRD42022287613

Review question

The objective of our study is to answer the following two research questions: 1. Among individuals with suspected TB and negative results with SSM, Xpert or Xpert Ultra (bacteriologically-negative TB suspects), what proportion receive TB treatment on clinical grounds, and how does this proportion differ systematically as a function of individual characteristics, clinical presentation, or other test results?

2. How has clinical diagnosis of bacteriologically-negative TB suspects changed over time with the increasing availability and use of Xpert, and do practices differ between countries and clinical settings?

Searches

[Source]: MEDLINE / PubMed, Embase, Cochrane Central Register of Controlled Trials

[Search dates]: published between 2010-2022 (in order to restrict to TB diagnostics using cartridge-based or other novel PCR-based diagnosis among TB suspects, using earliest Xpert publication in 2010 as the starting point)

Search strategy in PubMed:

("Tuberculosis"[Mesh:NoExp] OR "Tuberculosis, Pulmonary"[Mesh] OR tuberculosis[tiab])

AND

("Molecular Diagnostic Techniques"[Mesh:NoExp] OR ("Sputum"[Mesh] AND "Microscopy"[Mesh]) OR genexpert[tiab] OR xpert[tiab] OR smear microscopy[tiab] OR sputum microscopy[tiab])

AND

("randomized controlled trial"[pt] OR "controlled clinical trial"[pt] OR "random allocation"[mesh] OR "clinical trial"[pt] OR "evaluation study"[pt] OR "clinical trial"[tiab] OR random*[tiab] OR accuracy [tiab] OR evaluation[tiab])

AND

2010[pdat]:2022[pdat]

Types of study to be included

Randomized controlled trials, clinical trials, and evaluation studies will be included. Crossover studies will be excluded.

Condition or domain being studied

Currently used TB diagnostics are known to have imperfect sensitivity. For this reason, a negative result for current 1st-line diagnostics (sputum smear microscopy (SSM), Xpert, or Xpert Ultra) does not conclusively rule out TB. Evidence from programmatic settings suggests that a substantial fraction of TB diagnoses are made clinically, without bacteriological confirmation. It is useful to understand current practices around clinical diagnosis of TB as higher sensitivity diagnostics become more commonly used, as these diagnoses affect the overall sensitivity and specificity of the TB diagnostic algorithm, and the incremental impact of any new diagnostics that are introduced.

Participants/population

Studies eligible for the review include randomized control studies or cohort studies with data for TB suspects where (a) treatment decisions were based on a negative bacteriological test result (results of investigational tests not used for clinical decision-making will be ignored), and (b) with at least 1 week of clinical follow-up recording whether or not treatment was initiated. Study participants younger than 18 as well as population in low TB burden settings will be excluded.

Intervention(s), exposure(s)

Exposure includes identification as a TB suspect in a healthcare clinic, and receiving a negative result on a bacteriological test. Exposure excludes active case finding for TB.

Comparator(s)/control

NA

Context

We will collate multiple patient datasets from pragmatic trials of Xpert and Xpert Ultra in high-burden settings. Studies eligible for the review include randomized control studies or cohort studies with data for TB suspects where (a) treatment decisions were based on a negative bacteriological test result (results of investigational tests not used for clinical decision-making will be ignored), and (b) with at least 1 week of clinical follow-up recording whether or not treatment was initiated.

Main outcome(s)

The primary outcome of interest is the fraction of patients with suspected TB who are initiated on treatment among those who receive a negative SSM, Xpert, or Ultra result. By identifying how this proportion differ systematically as a function of individual characteristics, clinical presentation, or other test results, we can investigate the criteria clinicians use to treat for TB in the absence of a bacteriological diagnosis of TB.

Additional outcome(s)

Our secondary outcome of interest is the fraction of bacteriologically-negative TB suspects who are initiated on treatment by country, as a function of time. By investigating the trend within each country, we will be able to identify how the clinical diagnosis of bacteriologically-negative TB suspects changed over time with the increasing availability and use of Xpert, and how the practices differ between countries and clinical settings.

Data extraction (selection and coding)

First we will select pragmatic trial studies of Xpert and Xpert Ultra in high-burden settings based on the eligibility criteria and potentially include the variables stated below. De-identified patient-level data will be obtained by contacting authors/principal investigators of pragmatic trial studies and signing data use agreements (DUA).

Studies eligible for the review include randomized control studies or cohort studies with data for TB suspects where (a) treatment decisions were based on a negative bacteriological test result (results of investigational tests not used for clinical decision-making will be ignored), and (b) with at least 1 week of clinical follow-up recording whether or not treatment was initiated. We will extract 1) individual-level variables including the type of test received (Xpert, Ultra, SSN, culture), age, sex, relevant symptoms, results for any non-bacteriological tests performed, HIV status, and morbidity score if applicable, 2) setting-specific variables such as year, country, region, type of the clinic (primary, secondary, etc.) where the patient was evaluated, and 3) other variables including diagnosis result, treatment status, date of treatment initiation, date of testing, date of culture result returned (if applicable), and duration of follow-up.

Risk of bias (quality) assessment

There is a potential risk of bias in estimating the exposure (TB diagnosis) and outcome (decision to treat and treatment status within 1 week). If clinicians received results of other tests (different from standard of care) during decision making process, there exists a risk of bias. We will assess the risk of bias by examining the dates of diagnosis and treatment decision.

Strategy for data synthesis

We will construct logistic regression models to identify factors associated with the decision to treat for TB despite the negative test result. We will test for statistical heterogeneity (e.g., I^2) as part of the analysis, and in case of high heterogeneity, the analytic approach that accounts for the study level factors will be adopted. Analysis will be performed in R.

Analysis of subgroups or subsets

We plan to investigate how the practices differ by clinical symptoms, HIV status, and patient demographics. Thus we will stratify data by the relevant variables. We also plan to investigate how the practices differ between countries and clinical settings. Therefore, subgroups will consist of each country, and the fraction of bacteriologically-negative TB suspects who are initiated on treatment by country, as a function of time, will be investigated. This longitudinal analysis will be conducted in R. By investigating the trend within each country, we will be able to identify how the clinical diagnosis of bacteriologically-negative TB suspects changed over time with the increasing availability and use of Xpert, and how the practices differ between countries and clinical settings.

Contact details for further information

Sun Kim

sunkim1@hsph.harvard.edu

Organisational affiliation of the review

Harvard T.H. Chan School of Public Health

Review team members and their organisational affiliations [1 change]

Sun Kim. Harvard T.H. Chan School of Public Health

Dr Melike Can. Harvard T.H. Chan School of Public Health

Dr Tefera Agizew. U.S. Centers for Disease Control and Prevention, Botswana

Dr Andrew Auld. U.S. Centers for Disease Control and Prevention, Lusaka, Zambia.

Dr Maria Balcells. Infectious Disease Department, School of Medicine, Pontificia Universidad Católica de Chile.

Dr Stephanie Bjerrum. Department of Clinical Research, University of Southern Denmark, Odense Denmark.

Dr Keertan Dheda. Centre for Lung Infection and Immunity, Division of Pulmonology, Department of Medicine and UCT Lung Institute, Cape Town, South Africa.

Dr Susan Dorman. Medical University of South Carolina

Dr Aliasgar Esmail. Centre for Lung Infection and Immunity, Division of Pulmonology, Department of Medicine and UCT Lung Institute, Cape Town, South Africa.

Dr Katherine Fielding. TB Centre, Department of Infectious Disease Epidemiology, London School of Hygiene and Tropical Medicine, London, UK.

Dr Alberto Garcia-Basteiro. ISGlobal, Hospital Clínic – Universitat de Barcelona, Barcelona, Spain.

Dr Colleen Hanrahan. Epidemiology Department, Johns Hopkins Bloomberg School of Public Health, Baltimore, MD, USA.

Dr Wakjira Kebede. School of Medical Laboratory Sciences, Jimma University, Jimma Ethiopia.

Dr Mikashmi Kohli. FIND, the global alliance for diagnostics

Dr Anne Luetkemeyer. University of California San Francisco, San Francisco, CA, USA.

Carol Mita. Countway library, Harvard Medical School

Dr Byron Reeve. DSI-NRF Centre of Excellence for Biomedical Tuberculosis Research and SAMRC Centre for Tuberculosis Research, Division of Molecular Biology and Human Genetics, Faculty of Medicine and Health Sciences, Stellenbosch University, Tygerberg, South Africa.

Dr Denise Silva. Faculdade de Medicina, Universidade Federal do Rio Grande do Sul, Porto Alegre, Brazil.

Dr Sedona Sweeney. Faculty of Public Health and Policy, London School of Hygiene & Tropical Medicine, London, UK.

Dr Grant Theron. DSI-NRF Centre of Excellence for Biomedical Tuberculosis Research and SAMRC Centre for Tuberculosis Research, Division of Molecular Biology and Human Genetics, Faculty of Medicine and Health Sciences, Stellenbosch University, Tygerberg, South Africa.

Dr Anete Trajman. Federal University of Rio de Janeiro, Rio de Janeiro, Brazil.

Dr Anna Vassall. London School of Hygiene & Tropical Medicine

Dr Joshua Warren. Department of Biostatistics, Yale School of Public Health, New Haven, CT, USA.

Dr Marcel Yotebieng. Division of General Internal Medicine, Department of Medicine, Albert Einstein College of Medicine, New York City, NY, USA.

Dr Ted Cohen. Yale School of Public Health

Dr Nick Menzies. Harvard T.H. Chan School of Public Health

Type and method of review

Individual patient data (IPD) meta-analysis, Systematic review

Anticipated or actual start date

10 January 2022

Anticipated completion date [1 change]

31 May 2024

Funding sources/sponsors

We are supporting this research via a NIH-funded U01.

Grant number(s)

State the funder, grant or award number and the date of award

NIH funded grant number is 1U01AI152084-01, awarded on June 4th, 2020.

Conflicts of interest

Language

English

Country

United States of America

Published protocol

https://www.crd.york.ac.uk/prospero/display_record.php?ID=CRD42022287613

Stage of review [1 change]

Review Completed not published

Subject index terms status

Subject indexing assigned by CRD

Subject index terms

Humans; Sputum; Tuberculosis, Pulmonary

Date of registration in PROSPERO

07 February 2022

Date of first submission

08 January 2022

Stage of review at time of this submission [1 change]

Stage	Started	Completed
Preliminary searches	Yes	Yes
Piloting of the study selection process	Yes	Yes
Formal screening of search results against eligibility criteria	Yes	Yes
Data extraction	Yes	Yes
Risk of bias (quality) assessment	Yes	Yes
Data analysis	Yes	Yes

Revision note

The record has been updated following the completion of the review and the addition of new team members.

The record owner confirms that the information they have supplied for this submission is accurate and complete and they understand that deliberate provision of inaccurate information or omission of data may be construed as scientific misconduct.

The record owner confirms that they will update the status of the review when it is completed and will add publication details in due course.

Versions

07 February 2022

07 February 2022

02 July 2024
