# Supplementary material for: Factors associated with tuberculosis treatment initiation among bacteriologically negative individuals evaluated for tuberculosis: An individual patient data meta-analysis
Source: PLoS Med. 2025 Jan 13;22(1):e1004502. doi: 10.1371/journal.pmed.1004502 (PMC11729971; doi:10.1371/journal.pmed.1004502)
Supplement: Attachment — Submitted filename: Response-to-reviewers.docx [file pmed.1004502.s003.docx]

**Itemized response for PMEDICINE-D-24-01238R1**

**"Factors associated with tuberculosis treatment initiation among bacteriologically negative individuals evaluated for tuberculosis: an individual patient data meta-analysis”**

**Editorial comments:**

The editorial team read this manuscript with interest and thank the authors for choosing to submit it to PLOS Medicine. However, we agree with the reviewers’ comments and with those of the Academic Editor. Please note that these will need to be addressed in full to process to the next stage in the process towards publication.

1. Data Availability:
   PLOS Medicine requires that the de-identified data underlying the specific results in a published article be made available, without restrictions on access, in a public repository or as Supporting Information at the time of article publication, provided it is legal and ethical to do so. Please see the policy at http://journals.plos.org/plosmedicine/s/data-availability
   If the data are not freely available, please describe briefly the ethical, legal, or contractual restriction that prevents you from sharing it. Please also include an appropriate contact (web or email address) for inquiries (this cannot be a study author).

Response: Many of the datasets included in the analysis are restricted by IRB and require data use agreements to access and are therefore not freely available. We have edited the data availability statement to reflect this and have included information in the manuscript describing the contact information accessing for each dataset:

*“DATA AVAILABILITY. The data included in this study are restricted by IRB and require data use agreements for access, and therefore are not freely available. To access each dataset, please contact the respective authors listed in* ***Table S9*** *(see Supplement).” [page 19, lines 4-7]*

**Table S9. Contact information for accessing each dataset included in the study.**

| Study | Contact information for data access^*^ |
| --- | --- |
| Churchyard et al., 2015 | katherine.fielding@lshtm.ac.uk |
| Penn-Nicholson et al., 2021 | mikashmi.kohli@finddx.org |
| Dorman et al., unpublished | dorman@musc.edu |
| Dorman et al., 2018 | dorman@musc.edu |
| Theron et al., unpublished | gtheron@sun.ac.za |
| Mupfumi et al., 2014 | lmupfumi@gmail.com |
| Pereira et al., 2020 | drsilva@hcpa.edu.br |
| Hanrahan et al., 2015 | chanrah1@jhmi.edu |
| Mishra et al., 2020 | gtheron@sun.ac.za |
| Luetkemeyer et al. 2016 | annie.luetkemeyer@ucsf.edu |
| Bjerrum et al., 2020 | steph@medicinsk.dk |
| Agizew et al., 2019 | tefera.agizew@apopo.org |

*Subject to IRB approval and data use agreement.

2. Reporting guidance:
   Please report your study according to the relevant guidance which can be found here <https://www.equator-network.org/reporting-guidelines/>

Response: We have confirmed that reporting follows the PRISMA reporting guidelines and have included the PRISMA checklist with the supplementary materials.

3. Statistical reporting: (Please note that not all of this guidance will apply.)
   Please quantify the main results with 95% CIs and p values.
   When reporting p values please report as <0.001 and where higher as p=0.002, for example. When reporting 95% CIs please separate upper and lower bounds with commas instead of hyphens as the latter can be confused with reporting of negative values.
   Please include the actual amounts and/or absolute risk(s) of relevant outcomes (including NNT or NNH where appropriate), not just relative risks or correlation coefficients. (example for absolute risks: PMID: 28399126).
   Please include any important dependent variables that are adjusted for in the analyses.

Response: CIs are now separated with commas instead of hyphens, and absolute risks of relevant outcomes are added in **Table S8** (also copied below).

**Table S8. Relative and absolute change in probability^*^ of treatment initiation**

|  | Relative change (%)  in probability of treatment initiation  (95% Credible Intervals) | Absolute change^**^  in probability of treatment initiation  (95% Credible Intervals) |
| --- | --- | --- |
| Age category |  |  |
| 18 – 30 years | Ref | Ref |
| 31 – 40 years | 16.4 (-7.8, 48.6) | 0.5 (-0.2, 1.5) |
| 41 years and above | 10.6 (-12.6, 41.1) | 0.3 (-0.4, 1.3) |
| Sex |  |  |
| Female | Ref | Ref |
| Male | 58.0 (29.7, 89.3) | 1.8 (0.9, 2.8) |
| History of prior TB |  |  |
| None | Ref | Ref |
| Yes | 34.5 (5.8, 69.1) | 1.1 (0.2, 2.2) |
| Unknown | -26.4 (-71.4, 61.7) | -0.8 (-2.2, 1.9) |
| Reported cough |  |  |
| None | Ref | Ref |
| Yes | 314.7 (217.8, 437.7) | 9.9 (6.9, 13.8) |
| Reported night sweats |  |  |
| None | Ref | Ref |
| Yes | 47.7 (20.2, 84.8) | 1.5 (0.6, 2.7) |
| Reported fever |  |  |
| None | Ref | Ref |
| Yes | 12.5 (-8.7, 37.3) | 0.4 (-0.3, 1.2) |
| HIV |  |  |
| Negative | Ref | Ref |
| Positive, not on ART | 64.5 (22.1, 122.7) | 2.0 (0.7, 3.9) |
| Positive, on ART | -9.7 (-35.3, 28.8) | -0.3 (-1.1, 0.9) |
| Unknown | -17.5 (-44.2, 19.2) | -0.6 (-1.4, 0.6) |
| Diagnostic test |  |  |
| Sputum Smear | Ref | Ref |
| Xpert | -22.4 (-37.2, -3.9) | -0.7 (-1.2, -0.1) |
| Xpert Ultra | -42.2 (-69.3, 6.8) | -1.3 (-2.2, 0.2) |
| Year | -18.5 (-25.4, -9.7) | -0.6 (-0.8, -0.3) |

*The values are derived from the main analysis results (Table 2) using a base probability of 3.15%, as detailed in Table 1.

** The units are percentage points.

4. Prespecified analysis plan/study protocol:
   Did your study have a prospective protocol or analysis plan? Please state this (either way) early in the Methods section.

Response: The study protocol was registered with PROSPERO (CRD42022287613). This appears in the first paragraph in the Methods section (page 6, line 16).

a) If a prospective analysis plan (from your funding proposal, IRB or other ethics committee submission, study protocol, or other planning document written before analyzing the data) was used in designing the study, please include the relevant prospectively written document with your revised manuscript as a Supporting Information file to be published alongside your study, and cite it in the Methods section. A legend for this file should be included at the end of your manuscript.

Response: The protocol is now included as a supporting information file and is cited in the Methods section (page 6, line 16).

b) If no such document exists, please make sure that the Methods section transparently describes when analyses were planned, and when/why any data-driven changes to analyses took place.

Response: Not applicable (the protocol is included as a supporting information file).

c) In either case, changes in the analysis-- including those made in response to peer review comments-- should be identified as such in the Methods section of the paper, with rationale.

Response: Any changes are detailed in the methods.

5. Abstract layout:
   Please structure your abstract using the PLOS Medicine headings (Background, Methods and Findings, Conclusions).

Response: We have confirmed that the abstract uses the PLOS Medicine headings.

6. Author summary:
   At this stage, we ask that you include a short, non-technical Author Summary of your research to make findings accessible to a wide audience that includes both scientists and non-scientists. The authors summary should consist of 2-3 succinct bullet points under each of the following headings:
   • Why Was This Study Done? Authors should reflect on what was known about the topic before the research was published and why the research was needed.
   • What Did the Researchers Do and Find? Authors should briefly describe the study design that was used and the study’s major findings. Do include the headline numbers from the study, such as the sample size and key findings.
   • What Do These Findings Mean? Authors should reflect on the new knowledge generated by the research and the implications for practice, research, policy, or public health. Authors should also consider how the interpretation of the study’s findings may be affected by the study limitations. In the final bullet point of ‘What Do These Findings Mean?’, please describe the main limitations of the study in non-technical language.
   The Author Summary should immediately follow the Abstract in your revised manuscript. This text is subject to editorial change and should be distinct from the scientific abstract. Please see our author guidelines for more information: <https://journals.plos.org/plosmedicine/s/revising-your-manuscript#loc-author-summary>

Response: Thank you for this request. We have drafted an Author Summary and included this in the manuscript document:

*“****AUTHOR SUMMARY***

*Why Was This Study Done?*

- *Tuberculosis (TB) remains one of the leading causes of infectious disease death worldwide.*
- *Despite advancements in TB diagnostics, many diagnoses are still based on clinical judgement rather than bacteriological evidence.*
- *Understanding why clinicians decide to initiate TB treatment despite negative bacteriological test results can improve diagnostic accuracy and treatment outcomes.*

*What Did the Researchers Do and Find?*

- *We conducted a systematic review and meta-analysis of individual patient data from studies conducted between January 2010 and December 2022, where individuals were evaluated for TB.*
- *Key factors associated with initiating TB treatment after a negative bacteriological test included male sex, history of prior TB, reported cough and night sweats, and having HIV infection but not on antiretroviral therapy.* *Clinicians were less likely to initiate treatment if the initial test was a PCR-based diagnostic like Xpert MTB-RIF (as compared to sputum smear microscopy).*

*What Do These Findings Mean?*

- *The study identifies several factors that influence clinicians’ decisions to treat for TB despite negative bacteriological test results.*
- *These findings can help refine TB diagnostic and treatment protocols, improving patient outcomes and enhancing public health strategies.” [page 4, lines 1-22]*

**Reviewer #1:**
The authors undertook an individual-participant meta-analysis for evaluating factors associated with treatment initiation among patients with negative tests for tuberculosis. The manuscript is well written and I only have a few comments.

7. The authors should provide a more detailed discussion on how rates of treatment initiation might differ between those observed within clinical trials, observational studies and routine clinical practice. Currently it is only briefly touched upon in the limitations section.

Response: Thank you for this comment. There is limited evidence from routine settings on individuals receiving negative TB diagnoses at different points in the diagnostic algorithm (required to calculate treatment initiation rates). However, we agree with the reviewer that these rates may well differ from trial settings. We have added text to the discussion elaborating this point more fully, including a discussion of the factors which may drive differences in treatment initiation rates between routine and trial settings:

*“For example, it is possible that rates of clinical diagnosis will be lower in trial settings, if clinicians believe that missed diagnoses can be resolved through additional diagnostic testing undertaken as part of the trial (such as via sputum culture, performed in the majority of studies included in our review). If no additional testing is expected, clinicians may be more willing to make clinical diagnoses.” [page 18, lines 3-7]*

8. Discuss in more detail the differences in treatment initiation between various countries.

Response: Thank you for this suggestion, we have added text expanding on this point in the Discussion:

*“However, these setting-specific data were not available for analysis, contributing to differences in treatment initiation across various countries. These setting-specific differences were substantial (quantified by the country random effects included in the main analysis), pointing to the existence of additional determinants of treatment initiation not captured by our analysis. Additional research to identify these factors is needed.” [page 17, lines 16-20]*

9. How did the authors account for multiple testing using the same test (e.g. studies that used only one sputum Xpert vs. studies that tested 3 sputum samples with Xpert). Discuss how this may influence treatment initiation among those negative.

Response: In each of the studies included in the meta-analysis, clinicians only received the results of one initial TB test, and so the issue raised (clinicians having access to multiple Xpert results) did not arise. In some trials several Xpert tests were conducted, but only the results of one of these tests was shared with the clinician. However, most trials also collected samples for culture testing. It is possible that knowledge of this could have affected clinical behavior, and we have added text to the Discussion describing this.

*“For example, it is possible that rates of clinical diagnosis will be lower in trial settings, if clinicians believe that missed diagnoses can be resolved through additional diagnostic testing undertaken as part of the trial (such as via sputum culture, performed in the majority of studies included in our review). If no additional testing is expected, clinicians may be more willing to make clinical diagnoses. Conversely, for routine settings where follow-up testing is common (or where multiple tests are conducted concurrently) rates of clinical diagnosis could be similar to those observed in trial settings.” [page 18, lines 3-9]*

**Reviewer #2:**
Dear authors
With pleasure I read your manuscript. It reads very well.
Please find some recommendations:
Abstract:

10. Background: "after a negative diagnostic test result." please consider "after a negative bacteriological diagnostic test result. This will show you do not consider other tests, such as CXR.

Response: Thank you, we have edited this text:

*“…after a negative bacteriological test result…” [page 3 line 3]*

11. "Understanding factors associated with clinicians' decisions to initiate treatment for individuals with negative test results is critical for predicting the potential impact of new diagnostics." Is not that self-explanatory to me. I would keep this for the main text. In the intro I would emphasize the knowledge gap, not the potential impact.

Response: We have revised this text to increase clarity and emphasize the knowledge gap:

*"There is limited information on the factors that determine clinicians’ decisions to initiate TB treatment when initial bacteriological test results are negative." [page 3, lines 3-5]*

Main text
Intro:

12. "Compared to SSM and culture": no true comparison is made. Please reword.

Response: We have rephrased this text:

*“These tests combine rapid turn-around time and high sensitivity, enabling timely and accurate TB diagnosis” [page 5, lines 10-11]*

13. "If some of the individuals testing false-negative on an initial bacteriological test are subsequently treated based on clinical criteria, this may reduce the incremental impact achieved by adopting a more sensitive diagnostic. However, the widespread use of clinical diagnosis may also increase the number of individuals incorrectly treated for TB and overlook cases of drug-resistant TB" Consider adding that the roll-out of a good -but still imperfect- test may delay diagnosis as clinicians may be hesitant to start TB treatment based on clinical criteria.

Response: We agree with the reviewer that this scenario could occur, but feel it is too hypothetical to include here as such a test was not considered in our study.

14. "be the sole option": I think this should be "the main option", as some patients (children, patients with EP TB) are bacteriologically diagnosed.

Response: Thank you, we have edited this text:

*“…clinical diagnosis may be the primary diagnostic approach” [page 5, line 25]*

Methods:

15. "The target population for this study was individuals evaluated for pulmonary TB disease in routine clinical settings, who had received a negative result on an initial diagnostic test (e.g., smear microscopy, Xpert MTB/RIF). We conducted a systematic review to identify datasets describing the individual characteristics as well as the outcome of TB diagnosis (i.e., whether or not TB treatment was initiated) for individuals in this target population." What if after an initial test a follow-up test was done? Then diagnosis is not based on clinical ground, neither on the initial test.

Response: We restricted our analysis to scenarios where a single initial bacteriological test was conducted for clinical decision-making (i.e., omitting any testing done for research purposes). We have added text to the discussion hypothesizing how the opportunity to conduct follow-up tests could change the frequency of clinical diagnosis:

*“If no additional testing is expected, clinicians may be more willing to make clinical diagnoses. Conversely, for routine settings where follow-up testing is common (or where multiple tests are conducted concurrently) rates of clinical diagnosis could be similar to those observed in trial settings.” [page 18, lines 6-9]*

16. The same comment applies to "(b) where treatment decisions were based on diagnostic tests in routine use in that setting (i.e., additional tests conducted for research purposes were not used)". In routine care patients are tested often repeatedly. Would such a patient or study be eligible, and which test result would be considered as the initial test? If you would exclude those with a repeat positive test, you could have some selection? If you allow studies where more than one test was, the meaning of having a neg bact test would not be the same, as patients would have a lower post-test prob of TB.

Response: In the trials included on our meta-analysis, only one test result was used for clinical decision-making, even if multiple samples were collected and tested for research purposes. Therefore, we do not anticipate that availability of multiple initial test results influenced clinical behavior. However, samples were typically collected for testing via sputum culture, and it is possible that awareness of this by clinicians could have affected treatment decisions. We have added text discussing these issues:

*“For example, it is possible that rates of clinical diagnosis will be lower in trial settings, if clinicians believe that missed diagnoses can be resolved through additional diagnostic testing undertaken as part of the trial (such as via sputum culture, performed in the majority of studies included in our review). If no additional testing is expected, clinicians may be more willing to make clinical diagnoses. Conversely, for routine settings where follow-up testing is common (or where multiple tests are conducted concurrently) rates of clinical diagnosis could be similar to those observed in trial settings.” [page 18, lines 3-9]*

17. "While some studies undertook additional investigational tests clinicians were blinded to these results." Unclear to me. Your outcome "whether or not an individual initiated TB treatment" does not show whether a clinician was blinded for any follow-up result.

Response: The investigational tests being blinded to clinicians were indicated in the original studies. We added this context in our paper:

*“While some studies undertook additional investigational tests that were not part of the routine care, clinicians were blinded to these results (recorded in each trial report).” [page 8, lines 17-18]*

18. The categorization of age suggests that children were excluded. Was this shown above, in the methods? If so, I missed it.

Response: The exclusion of pediatric TB is reported on page 7 line 9. We have now clarified this to give the age cutoff:

*“We also excluded study participants younger than 18 years of age.” [page 7, line 9-10]*

Results: very clear

Discussion: very clear

19. Please add a paragraph on "Understanding factors associated with clinicians' decisions to initiate treatment for individuals with negative test results is critical for predicting the potential impact of new diagnostics"

Response: Thank you for this suggestion. We have added additional text discussing these issues:

*“If it is true that clinicians are less likely to make a clinical diagnosis following a negative Xpert or Xpert Ultra result (vs. a negative result on SSM), this could have implications for the impact of these new diagnostic tests on overall algorithm performance. Earlier modelling studies have demonstrated that clinical diagnosis could reduce the incremental effects of Xpert introduction on algorithm sensitivity, assuming that negative Xpert and negative SSM results are treated the same way by clinicians. These effects could be magnified if clinicians are less likely to make clinical diagnoses following a negative Xpert, further reducing the overall impact of Xpert introduction on algorithm sensitivity, while at the same time increasing algorithm specificity. Given the urgency of increasing TB case detection, further research on these potential mechanisms—and how to optimally balance the trade-offs involved in TB diagnosis—is needed.” [page 16, line 21 – page 17, line 6]*

**Reviewer #3:**
General comments:
This study presents a individual participant data meta-analysis exploring factors that associate with a decision to commence treatment for TB when rapid diagnostic tests (smear microscopy and NAATs) are negative.
The analysis includes data from 12 clinical cohort and randomised clinical studies, with a large sample size that is largely representative of patients encountered in high TB burden settings.
The study provides a useful overview of factors that associate with and are therefore presumed to influence the clinical suspicion of TB and decision to give treatment. In this respect it provides a review of physician behaviour and a measure of pre-test clinical probability of a diagnosis of TB that may be useful to understand the utility of existing and novel diagnostics. One example is presented in the paper of the effect of a negative Xpert or Xpert-ultra test on the clinician's assessment of TB likelihood.

Specific comments:

20. As the data is collected from participants recruited to research studies, clinical decision making is likely to have been influenced by the operating study protocols. These protocols are likely to include many of he factors identified here as risk factors for TB and as such the risk of incorporation bias cannot be excluded. Whilst the dataset is large, it is therefore difficult to know how representative these findings are of routine clinical practice.

Response: Thank you for identifying this issue. We have edited this discussion section to include this point.

*“It is also possible that trial protocols may have influenced the factors considered during clinical diagnosis.”*

*[page 18, lines 9-10]*

21. A significant limitation of the analysis, acknowledged by the authors, is that it is not underpinned by evidence to support or refute the appropriateness of the decision to start or withhold treatment for TB. The authors talk about the importance of avoiding both over-treatment and under-treatment in the absence of confirmatory diagnostics, but this manuscript does not provide us with information to draw any conclusions in this regard. Supporting evidence in the form of either bacterial confirmation where this data was available or a clinical and radiological response to treatment when culture was negative could provide useful insight of what may or may not be important.

Response: Thank you for this comment. We acknowledge that our analysis does not provide direct evidence to support or refute the appropriateness of clinical treatment decisions. In this study, our defined scope was on identifying the factors that affected decision-making, rather than evaluating the outcomes of those decisions. We did not collect data that would allow us to undertake the analysis suggested by the reviewer. However, we recognize the importance of such an analysis, and believe it will be an important topic for future research.

22. Furthermore, although mycobacterial culture is not routinely performed in high burden setting, it would be interesting and relevant to know whether the decision to delay starting treatment (>7days) differed between sites where culture was and was not performed. From a clinical standpoint, it could be entirely reasonable to wait for culture results in cases with an intermediate probability of TB.

Response: The large majority of studies included in our analysis also collected samples for culture testing. As the reviewer notes, this could have affected clinical decision-making. We have included text discussing this issue:

*“For example, it is possible that rates of clinical diagnosis will be lower in trial settings, if clinicians believe that missed diagnoses can be resolved through additional diagnostic testing undertaken as part of the trial (such as via sputum culture, performed in the majority of studies included in our review). If no additional testing is expected, clinicians may be more willing to make clinical diagnoses.” [page 18, lines 3-7]*

**Reviewer #4:**
This paper is concerned with an interesting problem, the factors affecting the decision to treat TB following a negative test.

23. Are there any unmeasured confounders reported in the literature that were not available in this analysis?

Response: Thank you for this question. There are several potential confounders not consistently included in study data, and therefore not available for the analysis. We have expanded the discussion section text describing these issues:

*“There are several limitations to this study. First, we were not able to analyze all factors that potentially inform clinician decision making, due to differences in the covariates recorded in the study datasets. It is possible that additional individual characteristics—such as recent weight loss or reporting a known TB contact, or medical co-morbidities such as diabetes or other immunosuppressive conditions—may impact clinical decision-making but were not consistently captured in the study data. When x-ray results were available they were found to have a major impact on clinical decision-making, but x-ray was only performed in a minority of studies. Similarly, it is possible that factors related to the healthcare setting, differences in national guidelines or protocols, or the capabilities of clinicians performing diagnosis may influence rates of clinical diagnosis.” [page 17, lines 7-16]*

24. Also, are there any other factors that clinicians tend to utilise in the diagnosis process that may be hard to quantify and therefore include in the analysis?

Response: The nature of clinical diagnosis is such that clinicians may draw on a wide range of evidence when making treatment decisions. The new text quoted in the prior item gives examples of several potential factors.

25. Any other data completeness issues, especially regarding how representative are the present data of the overall picture?

Response: Apart from the issues described in responses above (and in the text now added to the manuscript discussing these issues), we are not aware of other concerns regarding data completeness.

26. The authors have identified 51 studies as eligible, but only obtained the data from 18, is this an issue regarding the generalisation of the findings?

Response: While we only obtained data from 18 out of the 51 eligible studies, we made significant efforts to ensure that this subset is representative of the broader pool. Importantly, the countries of the studies included in our analysis—such as South Africa, Uganda, India, Brazil, Peru, and Zimbabwe —are also among those where the excluded studies were conducted. This overlap helps in maintaining the geographical and contextual relevance of our findings.

27. The student-t priors reported on supplement page 32 seem like a reasonable choice. However, it is well known that the PSRF criterion can be misleading since it is necessary for its value to be close to 1 but this is not a sufficient indication of algorithmic convergence.
    I suggest that the authors add the effective sample size, a more reliable measure of the quality of the algorithm (and its potential issues), summarising how many of those 4000 draws are roughly independent samples from the posterior density of interest.

Response: Thank you for this suggestion. We have inspected the ESS estimates for the fitted models, and have also included these in the manuscript. The software we are using (Stan) reports two ESS measures—Bulk Effective Sample Size (Bulk ESS) and Tail Effective Sample Size (Tail ESS)—which describe the efficiency of sampling in the central mass and tails of the posterior distribution, respectively. For each of the parameter in our models the Bulk and Tail ESS measures are all above 1,000. We have added this information in the **supplement** (also copied below) and attached the following table for your reference.

*“We also evaluated the effective sample sizes (ESS) for all our models. Bulk ESS and Tail ESS quantify sampling efficiency in the bulk of the posterior distribution and the tails of this distribution, respectively. The ESS values for each coefficient estimate were consistently above 1000, indicating that the sampling process was efficient and that our posterior samples are reliable.” [supplement page 12, line 25 – page 13, line 4]*

|  | **Main analysis (logit)** | | |
| --- | --- | --- | --- |
|  | Estimate  (95% CI) | Bulk ESS | Tail ESS |
| Age category |  |  |  |
| 18 – 30 years |  |  |  |
| 31 – 40 years | 0.16 (-0.09, 0.41) | 3244 | 3057 |
| 41 years and above | 0.10 (-0.15, 0.35) | 3362 | 3078 |
| Sex |  |  |  |
| Female |  |  |  |
| Male | 0.48 (0.29, 0.67) | 4695 | 3132 |
| History of prior TB |  |  |  |
| None |  |  |  |
| Yes | 0.31 (0.07, 0.54) | 5152 | 3244 |
| Unknown | -0.30 (-1.22, 0.50) | 4803 | 2779 |
| Reported cough |  |  |  |
| None |  |  |  |
| Yes | 1.53 (1.24, 1.83) | 4993 | 2778 |
| Reported night sweats |  |  |  |
| None |  |  |  |
| Yes | 0.41 (0.19, 0.63) | 3575 | 2942 |
| Reported fever |  |  |  |
| None |  |  |  |
| Yes | 0.12 (-0.10, 0.34) | 4666 | 3283 |
| HIV |  |  |  |
| Negative |  |  |  |
| Positive, not on ART | 0.52 (0.19, 0.83) | 3545 | 3149 |
| Positive, on ART | -0.10 (-0.46, 0.26) | 3353 | 2752 |
| Unknown | -0.20 (-0.59, 0.19) | 4156 | 3249 |
| Diagnostic test |  |  |  |
| Sputum Smear |  |  |  |
| Xpert | -0.27 (-0.49, -0.04) | 4001 | 2930 |
| Xpert Ultra | -0.56 (-1.17, 0.05) | 3240 | 2869 |
| Year | -0.20 (-0.30, -0.11) | 2593 | 2741 |

28. The basic model seems like a reasonable choice. Did the authors try any alternatives as sensitivity analysis? Say using probit or cloglog links? Or perhaps ensemble-based like random forests or XGB?
    This would also help to settle unclear findings like the effect of a negative result on Xpert Ultra.

Response: Thank you for this suggestion, we have now added a sensitivity analysis using the probit model. Based on this secondary analysis, the magnitudes of the coefficients in the logit model are generally larger than those in the probit model, which is expected due to the different scales of the logistic and inverse-Normal transformations. However, the signs of the coefficients are consistent across both models, and the t-ratios (ratio of estimate to standard error) indicate that the same predictors are statistically significant in both models. We also performed Leave-One-Out (LOO) cross-validation for model comparison. The expected log pointwise predictive density (ELPD) difference between the two models suggested that the logit model has a slightly better fit (ELPD difference of 3.6), though this difference was not statistically significant (standard error of the difference was 64.5). We have added this information in methods and results sections accordingly, and included **Table S5** in the supplement.

*“Additionally, we conducted a sensitivity analysis using the probit model, complemented by cross-validation.” [Page 9, lines 3-4]*

*“Our sensitivity analysis results showed that our findings remain consistent across different models (logit vs. probit) with cross-validation confirming that our main model exhibits a better fit (****Table S5****).” [page 13, lines 16-18]*

**Table S5. Sensitivity analysis comparing Logit vs. Probit model^*^.**

|  | **Main analysis (logit)** | | | **Probit model** | | |
| --- | --- | --- | --- | --- | --- | --- |
|  | Estimate | Standard Error | t-ratio | Estimate | Standard Error | t-ratio |
| Age category |  |  |  |  |  |  |
| 18 – 30 years | Ref |  |  | Ref |  |  |
| 31 – 40 years | 0.16 | 0.13 | 1.23 | 0.07 | 0.06 | 1.31 |
| 41 years and above | 0.10 | 0.13 | 0.78 | 0.04 | 0.06 | 0.67 |
| Sex |  |  |  |  |  |  |
| Female | Ref |  |  | Ref |  |  |
| Male | 0.48 | 0.10 | 4.85 | 0.21 | 0.04 | 4.61 |
| History of prior TB |  |  |  |  |  |  |
| None | Ref |  |  | Ref |  |  |
| Yes | 0.31 | 0.12 | 2.61 | 0.14 | 0.06 | 2.53 |
| Unknown | -0.30 | 0.44 | -0.68 | -0.10 | 0.18 | -0.58 |
| Reported cough |  |  |  |  |  |  |
| None | Ref |  |  | Ref |  |  |
| Yes | 1.53 | 0.15 | 10.04 | 0.68 | 0.07 | 10.23 |
| Reported night sweats |  |  |  |  |  |  |
| None | Ref |  |  | Ref |  |  |
| Yes | 0.41 | 0.11 | 3.69 | 0.19 | 0.05 | 3.74 |
| Reported fever |  |  |  |  |  |  |
| None | Ref |  |  | Ref |  |  |
| Yes | 0.12 | 0.11 | 1.10 | 0.05 | 0.05 | 1.02 |
| HIV |  |  |  |  |  |  |
| Negative | Ref |  |  | Ref |  |  |
| Positive, not on ART | 0.52 | 0.17 | 3.14 | 0.24 | 0.08 | 3.20 |
| Positive, on ART | -0.10 | 0.18 | -0.57 | -0.02 | 0.09 | -0.29 |
| Unknown | -0.20 | 0.20 | -1.02 | -0.07 | 0.08 | -0.83 |
| Diagnostic test |  |  |  |  |  |  |
| Sputum Smear | Ref |  |  | Ref |  |  |
| Xpert | -0.27 | 0.12 | -2.31 | -0.12 | 0.05 | -2.29 |
| Xpert Ultra | -0.56 | 0.31 | -1.78 | -0.19 | 0.15 | -1.25 |
| Year | -0.20 | 0.05 | -4.14 | -0.09 | 0.02 | -4.07 |

* The expected log pointwise predictive density (ELPD) difference of 3.6 with a standard error of 64.5 suggests that the main analysis (logit model) has a slightly better fit. However, the difference between the two models is not statistically significant.

29. How representative are these data overall? PloS Medicine takes a global view on public health and therefore this is an important discussion point.

Response: The data collated for this analysis are representative of a range of world regions and high TB burden countries. We have included studies from diverse geographic areas to ensure that our findings are globally relevant and applicable. The fact that the included data were drawn from trial settings could raise questions about representativeness of routine TB care, and these potential issues are discussed in the Discussion section (also copied below).

*“Moreover, the clinics in which these studies were conducted may have been selected based on their capacity to participate in research, which may limit their representativeness of the general context of TB care.” [page 18, lines 11-13]*

30. Including a country or study specific random effect term sounds reasonable.
    Are there any reasons to assume that these countries may not be "similar" and therefore exchangeable, perhaps initiating a discussion on accounting for some systematic difference?

Response: The covariates assessed in our study represent many of the factors that clinicians will consider when making treatment decisions. However, it is certainly possible that additional local factors will impact decision-making. We have expanded the section of the Discussion describing these issues:

*“Similarly, it is possible that factors related to the healthcare setting, differences in national guidelines or protocols, or the capabilities of clinicians performing diagnosis may influence rates of clinical diagnosis. However, these setting-specific data were not available for analysis, contributing to differences in treatment initiation across countries. These setting-specific differences were substantial (quantified by the country random effects included in the main analysis), pointing to the existence of additional determinants of treatment initiation not captured by our analysis. Additional research to identify these factors is needed.” [page 17, lines 14-20]*

31. Why exclude multi-drug resistant TB which represents an important problem? Please discuss.

Response: We agree with the reviewer that MDR-TB represents a major challenge. For TB diagnosis, MDR-TB cases often require different diagnostic approaches, including drug susceptibility testing, which may not be uniformly available across all settings included in our study. Given the likely small sample size of individuals with MDR-TB, we decided to exclude this group as we did not think we would be able to collate sufficient data to draw meaningful conclusions.

**Comments from the Academic Editor:**
The Academic Editor was in agreement with the reviewers’ comments and recommended that these be addressed in full. He commented that, whilst the study was reasonably well done in many respects, that the insights offered were, at present, limited.

32. He suggested that it would be useful if the analyses could be more robust in order to provide further depth to the results and to address concerns about heterogeneity. He suggested that the authors consider stratified analyses across diagnostic tests and perhaps HIV, too.

Response: We appreciate the additional comments provided by the academic editor. To address concerns about heterogeneity, we have now conducted stratified analyses across different diagnostic tests and HIV status. These analyses have been added to the supplement (**Tables S6** and **S7**, shown below). The coefficient estimates in these stratified analyses are similar across HIV strata and diagnostic tests, suggesting that our main analysis results are consistent across these subgroups.

33. He also suggested considering alternative ways to assess the value of the different TB tests, e.g. by calculating absolute risk of being treated with different patient profiles (e.g. HIV status, cough, night sweats) and different diagnostics.

Response: Regarding alternative ways to assess the value of different predictors, we have calculated both relative and absolute risks of treatment initiation for different patient profiles. These results are included in the supplement (**Table S8**, shown below), providing additional information on how each predictor influences treatment decisions.

34. He also wondered whether there might be some potentially interesting results should you consider analyses regarding trade-offs, e.g. an Xpert Ultra result is equal to cough lasting more than 2 weeks + night sweats in determining whether healthcare workers initiate treatment or not.

Response: Lastly, we recognize the potential value of analyzing the trade-offs between diagnostic test results and clinical symptoms. We believe this goes beyond the scope of the current analysis (for example, as compared to SSM, Xpert and Xpert Ultra also provide evidence on drug resistance, so the trade-off with duration of cough is more complicated than the comparison of treatment initiation rates). We believe this is a priority subject for future research:

*“If it is true that clinicians are less likely to make a clinical diagnosis following a negative Xpert or Xpert Ultra result (vs. a negative result on SSM), this could have implications for the impact of these new diagnostic tests on overall algorithm performance. Earlier modelling studies have demonstrated that clinical diagnosis could reduce the incremental effects of Xpert introduction on algorithm sensitivity, assuming that negative Xpert and negative SSM results are treated the same way by clinicians. These effects could be magnified if clinicians are less likely to make clinical diagnoses following a negative Xpert, further reducing the overall impact of Xpert introduction on algorithm sensitivity, while at the same time increasing algorithm specificity. Given the urgency of increasing TB case detection, further research on these potential mechanisms—and how to optimally balance the trade-offs involved in TB diagnosis—is needed.” [page 16, line 21 – page 17, line 6]*

**Table S6. Stratified analysis by diagnostic tests**

|  | Main analysis  (95% Credible Intervals) | SSM  (95% Credible Intervals) | Xpert + Xpert Ultra  (95% Credible Intervals) |
| --- | --- | --- | --- |
| Age category |  |  |  |
| 18 – 30 years | Ref | Ref | Ref |
| 31 – 40 years | 1.17 (0.92, 1.51) | 1.10 (0.73, 1.64) | 1.24 (0.90, 1.73) |
| 41 years and above | 1.11 (0.87, 1.43) | 0.80 (0.54, 1.22) | 1.35 (0.98, 1.86) |
| Sex |  |  |  |
| Female | Ref | Ref | Ref |
| Male | 1.61 (1.31, 1.95) | 1.80 (1.33, 2.46) | 1.48 (1.15, 1.91) |
| History of prior TB |  |  |  |
| None | Ref | Ref | Ref |
| Yes | 1.36 (1.06, 1.73) | 1.50 (1.01, 2.17) | 1.29 (0.95, 1.76) |
| Unknown | 0.73 (0.28, 1.65) | . | 0.81 (0.30, 1.86) |
| Reported cough |  |  |  |
| None | Ref | Ref | Ref |
| Yes | 4.62 (3.42, 6.27) | 4.48 (2.78, 7.46) | 4.50 (1.10, 1.93) |
| Reported night sweats |  |  |  |
| None | Ref | Ref | Ref |
| Yes | 1.50 (1.21, 1.90) | 1.60 (1.13, 2.28) | 1.45 (1.10, 1.93) |
| Reported fever |  |  |  |
| None | Ref | Ref | Ref |
| Yes | 1.13 (0.91, 1.39) | 1.00 (0.72, 1.40) | 1.22 (0.93, 1.60) |
| HIV |  |  |  |
| Negative | Ref | Ref | Ref |
| Positive, not on ART | 1.68 (1.23, 2.32) | 1.74 (1.10, 2.84) | 1.63 (1.10, 2.61) |
| Positive, on ART | 0.90 (0.64, 1.30) | 1.04 (0.54, 1.92) | 0.88 (0.55, 1.36) |
| Unknown | 0.82 (0.55, 1.20) | 1.16 (0.61, 2.13) | 0.65 (0.39, 1.08) |
| Diagnostic test |  |  |  |
| Sputum Smear | Ref | . | . |
| Xpert | 0.77 (0.62, 0.96) | . | Ref |
| Xpert Ultra | 0.57 (0.30, 1.07) | . | 0.79 (0.44, 1.44) |
| Year | 0.81 (0.74, 0.90) | 0.77 (0.62, 0.93) | 0.81 (0.71, 0.91) |

**Table S7. Stratified analysis by HIV results^*^**

|  | Main analysis  (95% Credible Intervals) | HIV negative | HIV Positive,  not on ART | HIV Positive,  on ART |
| --- | --- | --- | --- | --- |
| Age category |  |  |  |  |
| 18 – 30 years | Ref | Ref | Ref | Ref |
| 31 – 40 years | 1.17 (0.92, 1.51) | 1.81 (0.97, 3.36) | 1.10 (0.77, 1.54) | 0.86 (0.49, 1.58) |
| 41 years and above | 1.11 (0.87, 1.43) | 1.65 (0.95, 2.90) | 1.11 (0.78, 1.58) | 0.85 (0.48, 1.55) |
| Sex |  |  |  |  |
| Female | Ref | Ref | Ref | Ref |
| Male | 1.61 (1.31, 1.95) | 2.03 (1.29, 3.28) | 1.66 (1.25, 2.21) | 1.60 (1.03, 2.45) |
| History of prior TB |  |  |  |  |
| None | Ref | Ref | Ref | Ref |
| Yes | 1.36 (1.06, 1.73) | 1.72 (1.04, 2.85) | 1.17 (0.81, 1.71) | 1.62 (0.98, 2.71) |
| Unknown | 0.73 (0.28, 1.65) | 0.34 (0.02, 2.40) | 1.70 (0.38, 5.71) | 0.91 (0.13, 4.10) |
| Reported cough |  |  |  |  |
| None | Ref | Ref | Ref | Ref |
| Yes | 4.62 (3.42, 6.27) | 14.24 (2.07, 305.0) | 5.49 (3.87, 7.83) | 2.13 (1.22, 3.79) |
| Reported night sweats |  |  |  |  |
| None | Ref | Ref | Ref | Ref |
| Yes | 1.50 (1.21, 1.90) | 1.66 (1.06, 2.65) | 1.36 (0.97, 1.93) | 1.67 (1.06, 2.67) |
| Reported fever |  |  |  |  |
| None | Ref | Ref | Ref | Ref |
| Yes | 1.13 (0.91, 1.39) | 0.64 (0.41, 1.00) | 1.63 (1.19, 2.26) | 1.13 (0.73, 1.77) |
| HIV |  |  |  |  |
| Negative | Ref | . | . | . |
| Positive, not on ART | 1.68 (1.23, 2.32) | . | . | . |
| Positive, on ART | 0.90 (0.64, 1.30) | . | . | . |
| Unknown | 0.82 (0.55, 1.20) | . | . | . |
| Diagnostic test |  |  |  |  |
| Sputum Smear | Ref | Ref | Ref | Ref |
| Xpert | 0.77 (0.62, 0.96) | 0.53 (0.25, 1.03) | 0.80 (0.58, 1.09) | 0.58 (0.33, 1.01) |
| Xpert Ultra | 0.57 (0.30, 1.07) | 0.62 (0.18, 2.09) | 0.85 (0.19, 3.49) | 0.30 (0.10, 0.89) |
| Year | 0.81 (0.74, 0.90) | 0.57 (0.44, 0.72) | 0.90 (0.76, 1.05) | 0.98 (0.81, 1.16) |

*HIV Unknown stratum effective sample sizes were too low, indicating posterior means and medians may be unreliable.

**Table S8. Relative and absolute change in probability^*^ of treatment initiation**

|  | Relative change (%)  in probability of treatment initiation  (95% Credible Intervals) | Absolute change^**^  in probability of treatment initiation  (95% Credible Intervals) |
| --- | --- | --- |
| Age category |  |  |
| 18 – 30 years | Ref | Ref |
| 31 – 40 years | 16.4 (-7.8, 48.6) | 0.5 (-0.2, 1.5) |
| 41 years and above | 10.6 (-12.6, 41.1) | 0.3 (-0.4, 1.3) |
| Sex |  |  |
| Female | Ref | Ref |
| Male | 58.0 (29.7, 89.3) | 1.8 (0.9, 2.8) |
| History of prior TB |  |  |
| None | Ref | Ref |
| Yes | 34.5 (5.8, 69.1) | 1.1 (0.2, 2.2) |
| Unknown | -26.4 (-71.4, 61.7) | -0.8 (-2.2, 1.9) |
| Reported cough |  |  |
| None | Ref | Ref |
| Yes | 314.7 (217.8, 437.7) | 9.9 (6.9, 13.8) |
| Reported night sweats |  |  |
| None | Ref | Ref |
| Yes | 47.7 (20.2, 84.8) | 1.5 (0.6, 2.7) |
| Reported fever |  |  |
| None | Ref | Ref |
| Yes | 12.5 (-8.7, 37.3) | 0.4 (-0.3, 1.2) |
| HIV |  |  |
| Negative | Ref | Ref |
| Positive, not on ART | 64.5 (22.1, 122.7) | 2.0 (0.7, 3.9) |
| Positive, on ART | -9.7 (-35.3, 28.8) | -0.3 (-1.1, 0.9) |
| Unknown | -17.5 (-44.2, 19.2) | -0.6 (-1.4, 0.6) |
| Diagnostic test |  |  |
| Sputum Smear | Ref | Ref |
| Xpert | -22.4 (-37.2, -3.9) | -0.7 (-1.2, -0.1) |
| Xpert Ultra | -42.2 (-69.3, 6.8) | -1.3 (-2.2, 0.2) |
| Year | -18.5 (-25.4, -9.7) | -0.6 (-0.8, -0.3) |

*The values are derived from the main analysis results (Table 2) using a base probability of 3.15%, as detailed in Table 1.

** The units are percentage points.
