# Supplementary material for: Factors associated with tuberculosis treatment initiation among bacteriologically negative individuals evaluated for tuberculosis: An individual patient data meta-analysis
Source: PLoS Med. 2025 Jan 13;22(1):e1004502. doi: 10.1371/journal.pmed.1004502 (PMC11729971; doi:10.1371/journal.pmed.1004502)
Supplement: Attachment — Submitted filename: Response-to-reviewers.docx [file pmed.1004502.s004.docx]

**Itemized response for PMEDICINE-D-24-01238R1**

**"Factors associated with tuberculosis treatment initiation among bacteriologically negative individuals evaluated for tuberculosis: an individual patient data meta-analysis”**

**COMMENTS FROM THE ACADEMIC EDITOR:**

The academic editor supported moving this manuscript forward to a minor revision outcome. However, he did make one request.

1. He asked that the authors provide absolute risk and risk difference for at least the main analysis. In Table S8, the authors appear to state that they have done this, but it appears that only percentage change and risk difference were given. He kindly requests to see the absolute risk (i.e. 15% of PLWH not on ART with negative test received TB treatment vs. 10% of HIV negative individuals). He added that there should be no reference for absolute risk.

Response: Thank you. We have updated **Table S8** (now **Table S9**, shown below) to include absolute risk and risk difference.

**Table S9. Absolute risk and risk difference of treatment initiation^*^**

|  | Absolute risk (%)  of treatment initiation  (95% Credible Intervals) | Risk difference^**^  (95% Credible Intervals) | Relative change (%)  in probability of treatment initiation |
| --- | --- | --- | --- |
| Age category |  |  |  |
| 18 – 30 years | 2.89 (0.11, 12.67) | Ref | Ref |
| 31 – 40 years | 3.35 (0.13, 14.47) | 0.45 (-0.19, 2.41) | 17.7 (-7.7, 50.2) |
| 41 years and above | 3.17 (0.12, 13.77) | 0.28 (-0.41, 1.81) | 11.2 (-12.6, 40.9) |
| Sex |  |  |  |
| Female | 2.55 (0.10, 10.56) | Ref | Ref |
| Male | 3.93 (0.16, 15.96) | 1.39 (0.06, 5.55) | 59.3 (31.5, 91.2) |
| History of prior TB |  |  |  |
| None | 3.00 (0.12, 12.87) | Ref | Ref |
| Yes | 3.99 (0.16, 16.93) | 0.99 (0.03, 4.39) | 35.9 (7.0, 70.8) |
| Unknown | 2.43 (0.09, 11.48) | -0.57 (-4.27, 1.74) | -19.2(-70.2, 57.5) |
| Reported cough |  |  |  |
| None | 1.17 (0.08, 4.52) | Ref | Ref |
| Yes | 4.90 (0.39, 17.80) | 3.73 (0.30, 13.32) | 348.7 (231.1, 497.2) |
| Reported night sweats |  |  |  |
| None | 2.65 (0.12, 10.96) | Ref | Ref |
| Yes | 3.84 (0.17, 15.60) | 1.19 (0.06, 4.84) | 49.0 (20.7, 82.6) |
| Reported fever |  |  |  |
| None | 3.00 (0.12, 12.96) | Ref | Ref |
| Yes | 3.35 (0.14, 14.35) | 0.35 (-0.22, 1.93) | 13.0 (-8.1, 37.0) |
| HIV |  |  |  |
| Negative | 2.69 (0.13, 12.08) | Ref | Ref |
| Positive, not on ART | 4.29 (0.23, 18.64) | 1.60 (0.08, 6.81) | 67.6 (20.7, 126.9) |
| Positive, on ART | 2.46 (0.12, 11.05) | -0.24 (-1.91, 0.74) | -8.0 (-36.5, 28.9) |
| Unknown | 2.24 (0.11, 10.16) | -0.45 (-2.61, 0.41) | -16.7 (-44.3, 20.4) |
| Diagnostic test |  |  |  |
| Sputum Smear | 3.75 (0.21, 16.96) | Ref | Ref |
| Xpert | 2.95 (0.16, 13.54) | -0.80 (-3.66, -0.02) | -22.0 (-37.0, -3.2) |
| Xpert Ultra | 2.32 (0.12, 11.16) | -1.43 (-6.80, 0.14) | -38.9 (-68.3, 8.8) |

* The values are derived using posterior draws of predicted probabilities from the main regression model (Table 2).

** The units are percentage points.

------------------------------------------------------------
**COMMENTS FROM THE REVIEWERS:**

**Reviewer #1:** Thank you for providing a revised version of the manuscript. The authors have fully addressed all my comments. I have no further comments
Response: Thank you.

**Reviewer #2:** Dear  authors
Almost all comments were addressed.

One of my comments might not have been clear.
First I show the history of our exchange, then I re-formulate my comment, with a few more words.

In the manuscript: "If some of the individuals testing false-negative on an initial bacteriological test are subsequently treated based on clinical criteria, this may reduce the incremental impact achieved by adopting a more sensitive diagnostic. However, the widespread use of clinical diagnosis may also increase the number of individuals incorrectly treated for TB and overlook cases of drug-resistant TB"

My comment was: Consider adding that the roll-out of a good -but still imperfect- test may delay diagnosis as clinicians may be hesitant to start TB treatment based on clinical criteria.

Your response was: We agree with the reviewer that this scenario could occur, but feel it is too hypothetical to include here as such a test was not considered in our study.

My follow-up comment: All TB diagnostic tests available at present are imperfect, especially with regards to sensitivity. Hence, my comment was not based on hypothetical grounds. Since the implementation of Xpert MTB/RIF, an imperfect test (with regards to its sensitivity, especially in some subgroups), I see more diagnostic delays, as clinicians are more hesitant to make a clinical decision. Hence, my comment still stands.
I agree with you that clinical decision making may mask the impact of the implementation of more sensitive diagnostics, and that timely clinical diagnosis of TB might reduce the possibility of diagnosing eg RR-TB. Indeed, a more exhaustive evaluation, which in decentralized setting often implies referral of the patient or transport of samples to a central lab, will result in more accurate diagnosis, including resistant TB. However, in patients with an initial negative TB test, and with underlying TB disease, such diagnostic delays also may result in more advanced TB disease, pre-treatment attrition, and continued transmission.

2. I therefore recommend to show a more balanced view on pro's and con's of clinical decision making for the diagnosis of TB.

Response: Thank you for clarifying this point (and sorry for misunderstanding the first time). We believe this point is best placed in the discussion, where we are discussing the consequences of there being a lower probability to make clinical diagnoses with Xpert. We have added the following text:

*“For individuals testing false-negative with Xpert, greater hesitance to initiate treatment based on clinical criteria could increase diagnostic delays, prolonging TB-related morbidity and mortality risks.” [page 18 line 10 – 13]*

**Reviewer #5:** This paper investigates the factors influencing clinicians' decisions to initiate tuberculosis (TB) treatment after a negative bacteriological test result, a situation that occurs in over one-third of global TB diagnoses. Using a systematic review and individual patient data meta-analysis, the study analyzed trials and cohort studies conducted from 2010 to 2022. It found that several factors increased the likelihood of treatment initiation, including male sex, a history of prior TB, symptoms like cough and night sweats, and HIV infection without ART. However, clinicians were less likely to start treatment following a negative result from more sensitive, PCR-based diagnostics like Xpert MTB/RIF, compared to traditional smear microscopy.

I believe the authors have effectively addressed the reviewers' comments and concerns. However, I do have a couple of additional questions.

3. Since Bayesian methods were used, I didn't notice any mention of the burn-in period for the posteriors. Could the authors clarify the burn-in they used?

Response: We thank the reviewer for raising this point. In our analysis, we discarded the first 1000 iterations as the burn-in period, to ensure the chains have reached stationarity before sampling from the posterior distribution. We have added this to the text:

*“During the sampling process we ran 4 chains for 2000 samples each, retaining the last 1000 samples of each chain to produce a final set of 4,000 posterior samples.” [appendix page 13, lines 22-24]*

4. Additionally, have the authors checked the trace plots of the coefficients to ensure that the estimates have fully converged?

Response: We have reviewed the trace plots, and they confirm full convergence of all estimates. We have added this to the text.

*“Convergence was assessed using Gelman-Rubin statistic (i.e., potential scale reduction factor (PSRF)), and by reviewing trace plots. PSRF values for all parameters were observed to be 1.00, confirming convergence (41), and trace-plots indicated chains were fully mixed.” [appendix page 13, lines 24-25 and page 14, lines1-2]*

------------------------------------------------------------
**REQUESTS FROM THE EDITORS:**
-       Thank you for providing a completed PRISMA checklist. Could you please revise the right most column, replacing the page numbers with paragraph/section and line numbers? This is because page numbers are subject to change during the publication process. Apologies for not highlighting this requirement beforehand.

Response: Thanks, they are updated now.

-       Thank you for providing us with an Author Summary. This section, as it stands, needs a small revision. In the final bullet point of ‘What Do These Findings Mean?’, please describe the main limitations of the study in non-technical language.

Response: Thanks, we added the limitations and copied below.

*“More evidence is needed on clinicians' decision-making processes, which we did not assess in this study.” [page 5, lines 3-4]*

-       In the last sentence of the Abstract Methods and Findings section, please describe the main limitation(s) of the study's methodology.

Response: We added the limitation of the study’s methodology and copied below.

*“We were not able assess why clinicians made treatment decisions, as these data were not available.” [page 3, lines 19-20]*

-       In your Abstract, please combine the Methods and Findings sections into one section: i.e. ‘Methods and findings’.

Response: Now they are edited.

-       Please add the following statement, or similar, to the Methods section: ‘This study is reported as per the Preferred Reporting Items for Systematic Reviews and Meta-Analyses (PRISMA) guideline (S1 Checklist).’

Response: Thanks, now the texts are added.

Additionally, on reviewing the revised manuscript, we realized it could be valuable to provide another sensitivity analysis that combined Xpert and Xpert Ultra into a single category of rapid diagnostic tests (RDTs), so these tests could be compared to smear as a single group. We have updated the methods and results sections accordingly, along with the table below (in the appendix). While we believe this additional sensitivity analysis adds value, if including this material raises any concerns we would be happy to remove it. Please let us know your thoughts.

**Table S8. Odds ratios of TB treatment initiation following a negative diagnostic test result (Xpert/ Xpert Ultra combined).**

|  | Univariable analysis  (95% Credible Intervals) | Multivariable analysis*  (95% Credible Intervals) |
| --- | --- | --- |
| Age category |  |  |
| 18 – 30 years | Ref | Ref |
| 31 – 40 years | 1.44 (1.13, 1.83) | 1.17 (0.91, 1.53) |
| 41 years and above | 1.42 (1.12, 1.80) | 1.10 (0.86, 1.43) |
| Sex |  |  |
| Female | Ref | Ref |
| Male | 1.80 (1.50, 2.16) | 1.61 (1.33, 1.96) |
| History of prior TB |  |  |
| None | Ref | Ref |
| Yes | 1.61 (1.13, 2.03) | 1.37 (1.08, 1.74) |
| Unknown | 0.81 (0.32, 1.77) | 0.73 (0.28, 1.69) |
| Reported cough |  |  |
| None | Ref | Ref |
| Yes | 5.93 (4.52, 7.88) | 4.60 (3.44, 6.18) |
| Reported night sweats |  |  |
| None | Ref | Ref |
| Yes | 2.34 (1.89, 2.89) | 1.51 (1.21, 1.89) |
| Reported fever |  |  |
| None | Ref | Ref |
| Yes | 1.84 (1.48, 2.28) | 1.13 (0.91, 1.39) |
| HIV |  |  |
| Negative | Ref | Ref |
| Positive, not on ART | 1.88 (1.38, 2.57) | 1.70 (1.23, 2.33) |
| Positive, on ART | 0.73 (0.52, 1.02) | 0.90 (0.63, 1.28) |
| Unknown | 0.98 (0.65, 1.46) | 0.82 (0.54, 1.22) |
| Diagnostic test |  |  |
| Sputum Smear | Ref | Ref |
| Xpert or Xpert Ultra | 0.56 (0.45, 0.69) | 0.77 (0.61, 0.96) |
| Year | 0.77 (0.71, 0.83) | 0.80 (0.73, 0.87) |

*Multivariable regression model also included country random effects, Ref = reference category.
